# Pioneer factor ASCL1 cooperates with the mSWI/SNF complex at distal regulatory elements to regulate human neural differentiation

**DOI:** 10.1101/gad.350269.122

**Published:** 2023-03-01

**Authors:** Oana Păun, Yu Xuan Tan, Harshil Patel, Stephanie Strohbuecker, Avinash Ghanate, Clementina Cobolli-Gigli, Miriam Llorian Sopena, Lina Gerontogianni, Robert Goldstone, Siew-Lan Ang, François Guillemot, Cristina Dias

**Affiliations:** 1 Neural Stem Cell Biology Laboratory, the Francis Crick Institute, London NW1 1AT, United Kingdom;; 2 Bioinformatics and Biostatistics Science and Technology Platform, the Francis Crick Institute, London NW1 1AT, United Kingdom;; 3 Medical and Molecular Genetics, School of Basic and Medical Biosciences, Faculty of Life Sciences and Medicine, King's College London, London SE1 9RT, United Kingdom

**Keywords:** pioneer transcription factor, chromatin regulation, ASCL1, mSWI/SNF, ChIP-seq, ATAC-seq, scRNA-seq, neural stem cell, neurogenesis

## Abstract

Pioneer transcription factors are thought to play pivotal roles in developmental processes by binding nucleosomal DNA to activate gene expression, though mechanisms through which pioneer transcription factors remodel chromatin remain unclear. Here, using single-cell transcriptomics, we show that endogenous expression of neurogenic transcription factor ASCL1, considered a classical pioneer factor, defines a transient population of progenitors in human neural differentiation. Testing ASCL1's pioneer function using a knockout model to define the unbound state, we found that endogenous expression of ASCL1 drives progenitor differentiation by *cis*-regulation both as a classical pioneer factor and as a nonpioneer remodeler, where ASCL1 binds permissive chromatin to induce chromatin conformation changes. ASCL1 interacts with BAF SWI/SNF chromatin remodeling complexes, primarily at targets where it acts as a nonpioneer factor, and we provide evidence for codependent DNA binding and remodeling at a subset of ASCL1 and SWI/SNF cotargets. Our findings provide new insights into ASCL1 function regulating activation of long-range regulatory elements in human neurogenesis and uncover a novel mechanism of its chromatin remodeling function codependent on partner ATPase activity.

## Abstract (precis)

In this study, Păun et al. describe how pioneer transcription factor ASCL1 regulates activation of gene regulatory elements involved in neuronal differentiation. They also show that ASCL1 interacts with the ATPase-dependent mSWI/SNF complex, elucidating a new mechanism of chromatin remodeling in human neurogenesis.

A particular class of transcription factors (TFs) regulating the differentiation of tissues in the embryo controls early steps in the development of cell lineages via their pioneer activity (Cirillo et al. 2002; Zaret and Carroll 2011; Iwafuchi-Doi et al. 2016; Gao et al. 2019; Mayran et al. 2019; Song et al. 2021; Yu et al. 2021). Most TFs engage with their cognate binding sites in the genome only when in an open chromatin configuration, where DNA is not wrapped around nucleosomes. In contrast, pioneer transcription factors have the capacity to recognize binding sites in regulatory elements embedded in closed chromatin (i.e., in DNA wrapped around nucleosomes) and to open chromatin, thus facilitating the binding of other transcription factors and the transcription of the associated genes (Iwafuchi-Doi and Zaret 2014; Soufi et al. 2015). A third category has been proposed, “nonclassical” pioneer TFs, which remodel chromatin but have DNA binding properties constrained by nucleosome position and require the recruitment of chromatin remodeling complexes to their target sites (Minderjahn et al. 2020). Furthermore, the classical pioneer factor hypothesis has recently been challenged by the demonstration that nonpioneer transcription factors can display pioneering function when ectopically expressed in a concentration- and genomic context-dependent manner (Hansen and Cohen 2022; Hansen et al. 2022). However, the mechanisms by which endogenous transcription factors expressed at physiological concentration exert a putative pioneer activity have remained unexplored.

Neurogenesis is characterized by important cell state transitions that require epigenetic remodeling of chromatin in a tightly orchestrated fashion to ensure generation of neurons of distinct identities in appropriate numbers and at defined locations, a process that remains poorly understood in human cortical development. Here we examine the activity of the endogenous proneural pioneer factor ASCL1 in human neurogenesis. ASCL1 function has been studied mostly in the developing mouse brain (Nieto et al. 2001; Castro et al. 2011; Pacary et al. 2011; Andersen et al. 2014), where it was shown to regulate multiple steps of neurogenesis, including the proliferation and neuronal fate commitment of multipotent progenitors, and the differentiation and migration of postmitotic neurons (Tomita et al. 2000; Nieto et al. 2001; Castro et al. 2011; Pacary et al. 2011; Borromeo et al. 2014). However, ASCL1 expression has also been reported in progenitor cells in the human embryonic telencephalon (Hansen et al. 2010; Alzu'bi and Clowry 2019), suggesting that it has retained a function in the regulation of neurogenesis in humans. ASCL1 is a basic helix–loop–helix (bHLH) proneural TF, a class of TFs that has a prominent role in the regulation of neurogenesis (Bertrand et al. 2002; Huang et al. 2014) and has been shown to display pioneer activity, recognizing nucleosomal DNA enriched for a short E-box motif (Henke et al. 2009; Soufi et al. 2015).

Evidence for the pioneer activity of ASCL1 comes from in vitro reconstituted nucleosome binding assays and protein overexpression in cultured cells (Heinrich et al. 2010; Wapinski et al. 2013; Chanda et al. 2014; Raposo et al. 2015; Soufi et al. 2015; Park et al. 2017; Fernandez Garcia et al. 2019). In particular, ASCL1 has been shown to recognize its neuronal targets in a closed chromatin state when overexpressed in fibroblasts (initiating neuronal reprogramming), undifferentiated neural stem cells, and glioblastoma stem cells (Wapinski et al. 2013; Raposo et al. 2015; Park et al. 2017). However, the pioneer activity of the endogenous ASCL1 protein has not yet been investigated, nor has the mechanism by which local chromatin structure is affected by ASCL1 binding.

Different models have been proposed to explain the capacity of pioneer factors to open chromatin, including physical eviction of nucleosomes by the pioneer factor–DNA interaction (Cirillo et al. 2002; Michael et al. 2020) and/or interaction with other transcription factors and proteins with chromatin remodeling capacity (Hu et al. 2011; Theodorou et al. 2013; Wang et al. 2014; Takaku et al. 2016; King and Klose 2017; Cernilogar et al. 2019). The mammalian SWI/SWF (mSWI/SWF) complexes represent attractive candidates among chromatin remodelers to interact with ASCL1 because of their role in the regulation of neurogenesis, exemplified by their implication in multiple neurodevelopmental disorders (Kosho et al. 2014; Pulice and Kadoch 2016; Sokpor et al. 2017). While core subunits of BAF complexes are ubiquitously expressed, other subunits are incorporated with developmental stage and cell type specificity (Son and Crabtree 2014; Mashtalir et al. 2020). Importantly, changes in the combinatorial assembly of BAF complexes underpin the transition from neural progenitors to neurons in vivo and in vitro (Lessard et al. 2007; Yoo et al. 2009; Staahl et al. 2013), with specific subunits incorporating in progenitor-specific (npBAF) or neuronal-specific (nBAF) complexes. The developmental overlap of npBAF and proneural transcription factors was recently highlighted by the demonstration that conditional deletion of npBAF subunit ACTL6A in the mouse cortex leads to decreased chromatin accessibility at specific neural TF binding sites, including ASCL1 (Braun et al. 2021). We therefore hypothesized that the pioneer TF ASCL1 may interact with mBAF SWI/SNF complexes to regulate chromatin accessibility at neurogenic loci to coordinate neurodifferentiation.

In this study, we have investigated the mechanisms of pioneer transcription factor function in a pivotal cell state transition in human neurogenesis in a cell culture model of human cortical development. Using single-cell transcriptional resolution, we show that expression of proneural TF ASCL1 defines a cell population of transitional neural progenitors immediately preceding differentiation to neurons. ASCL1 has pioneer activity, binding regulatory elements implicated in neurogenic programs in a closed chromatin configuration and opening chromatin at many of its target sites. It also binds “permissive” sites with a low degree of accessibility in an ASCL1 naïve state to induce further chromatin changes. Cooperative mSWI/SNF ATPase-dependent remodeling activity is required for ASCL1 activity at a subset of its targets. Together, our findings support a model in which ASCL1 acts as a pioneer factor in distinct ways during human telencephalic neurogenesis. It acts as a classical pioneer transcription factor, binding heterochromatin for regulation of a fraction of its targets, largely without mSWI/SNF interaction. However, it also regulates chromatin accessibility at a large number of loci in the genome via cooperative binding with the mSWI/SNF chromatin remodelers at sites of permissive DNA.

## Results

### ASCL1 is expressed in cells transitioning from dividing progenitors to postmitotic neurons during human cortical neurogenesis in vitro and in vivo

**Figure 1.**
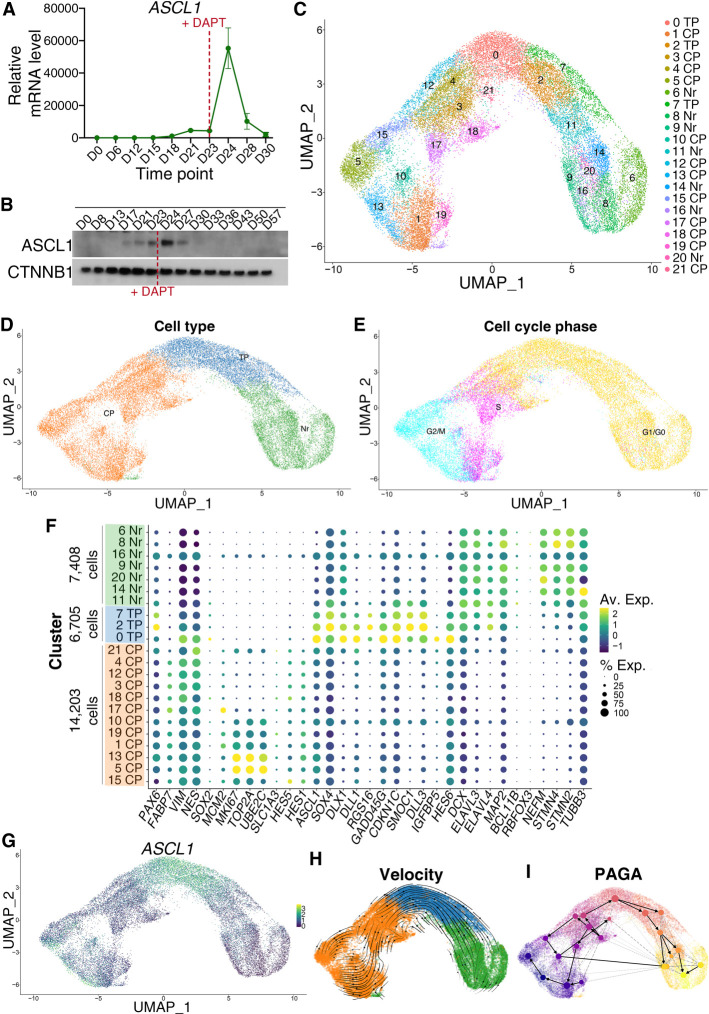
ASCL1 mRNA expression marks a transitional cell population bridging progenitors and postmitotic neurons. (*A*) qRT-PCR analysis of *ASCL1* expression at multiple time points during neural differentiation of human iPSCs; mRNA expression relative to DIV0 (D0). *ASCL1* shows a transient spike in expression at DIV24 (D24), following Notch inhibition (via DAPT addition, indicated by a red dashed line) at DIV23 (D23). Error bars represent mean ± SEM for three biological replicates. (*B*) Western blotting shows transient ASCL1 protein increase during neural differentiation. Notch inhibition is indicated by a red dashed line. CTNNB1 loading control is included. (*C*) Uniform manifold approximation and projection (UMAP) plot and unsupervised clustering of single-cell transcriptomes from 28,316 cells in DIV24 neural cultures treated with γ-secretase inhibitor DAPT, collected from three independent cultures. Dots represent single cells. Colors represent the different clusters identified at the *right*. (*D*) Clusters from *C* were grouped into three broad cellular state clusters based on gene expression of canonical markers (see *F*). A large cluster of *VIM*^+^*NES*^+^ cells uniquely enriched for *ASCL1* and its transcription targets, lacking in neuronal markers and positioned between a cluster of cycling progenitors (CPs) and a cluster of neurons (Nrs), was termed “transitional progenitors” (TPs). (*E*) Predicted cell cycle phases of cells based on their expression of cell cycle-related genes. Transitional progenitors are found in the G1/G0 phase. (*F*) Dot plot representation of the expression of genes used for post-hoc annotation of unsupervised clusters (shown in *C*) to classify cell state identities represented in *D*. Dot size indicates proportion of cells in each cluster expressing a gene, and shading indicates the relative level of gene expression according to the key at the *right*. (*G*) Single-gene expression overlaid onto UMAP plot defined in *C* shows *ASCL1* expression is enriched in transitional progenitors. (*H*) RNA velocity vectors projected onto the UMAP plot show differentiation directionality from cycling progenitors to neurons through transitional progenitors. A second direction was identified that reflected the cell cycle phases. (*I*) A partition-based graph abstraction (PAGA) velocity graph, with PAGA connectivities (dashed) and transitions (solid/arrows), shows a similar differentiation trajectory. The size of a node reflects the number of cells belonging to the corresponding cluster.

The function of ASCL1 as a pioneer TF has been established by studying the reprogramming of somatic cells into neurons (Wapinski et al. 2013, 2017; Chanda et al. 2014; Raposo et al. 2015), yet the role of endogenous ASCL1 in human cortical development remains unexplored. To investigate this, we modeled human cortical development in vitro by using a two-dimensional (2D) adherent dual-SMAD inhibition protocol to promote the differentiation of human induced pluripotent stem cells (iPSCs) into cortical neurons (modified from Chambers et al. 2009; Shi et al. 2012). We first characterized the expression of ASCL1 in this model system. We detected *ASCL1* transcript expression by qRT-PCR and protein expression by Western blotting at 17 d of differentiation in vitro (DIV) (Fig. 1A,B), preceding the onset of expression of neuronal markers *MAP2*, *HUC/D*, and *CTIP2* at DIV18–20 (Supplemental Fig. S1A). Addition of γ-secretase inhibitor DAPT at DIV23 accelerated and synchronized the cell cycle exit and differentiation of neural progenitor cells (Supplemental Fig. S1B,C; Crawford and Roelink 2007). ASCL1 RNA and protein levels increased rapidly and peaked 24 h after DAPT addition (DIV24), before declining and becoming undetectable after DIV30 (Fig. 1A,B). Concomitant with the burst in ASCL1 expression at DIV23–24, neural progenitors exposed to DAPT showed a rapid decrease in expression of the cell proliferation marker *MKi67*, up-regulation of cell cycle arrest genes *CDKN1C* and *GADD45G*, and strong up-regulation of neuronal markers *MAP2*, *HUC/D*, and *CTIP2* (Supplemental Fig. S1A,D).

To further investigate the concomitance of ASCL1 expression with cell cycle exit, we dissected the heterogeneity of neural lineage cell states in iPSC-derived neural cultures by performing a single-cell RNA-seq (scRNA-seq) analysis in DIV24 cultures treated with DAPT using the 10X Genomics Chromium single-cell platform. Unsupervised clustering of a data set of 28,316 cells collected from three concurrent differentiated cultures resulted in 22 clusters of cells (Fig. 1C; Supplemental Fig. S1E), which were then merged based on their expression of predefined canonical markers of the progenitor state (*SOX2*, *PAX6*, and *HES5*), proliferation (*MKi67* and *MCM2*), cell cycle exit (*CDKN1C* and *GADD45G*), and neuronal differentiation (*HES6*, *SOX4*, *DCX*, *MAP2*, *ELAVL4*, *GAD2*, and *SLC17A6).* This resulted in three main cell clusters containing, respectively, cycling neural progenitors, neurons, and transitional progenitors bridging the two previous clusters (Fig. 1D–F). The cluster defined as “transitional progenitors” coexpressed markers of progenitors (*VIM* and *NES*), cell cycle exit (*CDKN1C*, *GADD45G,* and *HES6*), and early neuronal differentiation (*HES6* and *SOX4*) with very low levels of proliferation markers (*MKi67* and *MCM2*) and of neuronal markers (*MAP2*, *NEFM*, *STNM2*, and *STMN4*) (Fig. 1F). Thus, this cluster represents progenitors transitioning between mitotic progenitors and differentiating neurons; i.e., likely preceding or just following the last progenitor cell division (Fig. 1D–F).

Differential gene expression analysis confirmed the transitional progenitor cluster is uniquely enriched for *ASCL1* (Fig. 1F,G), with low-level expression also found in a subset of cycling progenitors. This is reminiscent of observations in mice, where ASCL1 expression has an oscillating pattern in cycling neural progenitors, and its accumulation coincides with neuronal differentiation (Imayoshi et al. 2013). We also observed enrichment for several of its known transcriptional targets, including *SMOC1*, *RGS16*, and *IGFBP5* (Castro et al. 2011; Martynoga et al. 2012), supporting that it is a bona fide cell state defined by ASCL1 expression rather than a mixture of progenitors and neurons. To characterize the cell state transitions in our model of neural differentiation, we predicted directed dynamic transitions between clusters using RNA velocity analysis (with the scVelo tool) (Bergen et al. 2020). The estimated velocity vector streams delineate the direction of cell state transitions, with cycling progenitor cells at the apex and a trajectory toward transitional progenitors followed by postmitotic neurons (Fig. 1H). Further trajectory inference using PAGA (partition-based graph abstraction) was used to estimate connectivity and transition between groups of cells (Fig. 1I; Wolf et al. 2019), which, complemented by RNA velocity information, supported the directionality of the transitions between cell states. Together, our scRNA-seq analysis shows that during human neurogenesis, an increase in *ASCL1* expression specifically marks a transient population of progenitors differentiating into neurons.

To examine how these findings relate to in vivo neurodevelopment, we compared our data with publicly available scRNA-seq cluster profiles from developing first trimester fetal forebrains (Braun et al. 2022). Using cluster label transfer, we show that cell states present in vivo map to clusters in our in vitro system. Furthermore, not only are the cell states found in vitro similar to known cell states in vivo, but also the order of developmental stages in vitro follows the expected developmental path observed in vivo (Supplemental Fig. S1F). The fetal scRNA-seq clusters with highest correlation to in vitro transitional progenitors in terms of average expression profile correspond to neuroblasts and neuronal intermediate progenitor cell clusters (or cell states) (Supplemental Table S1).

**Figure 2.**
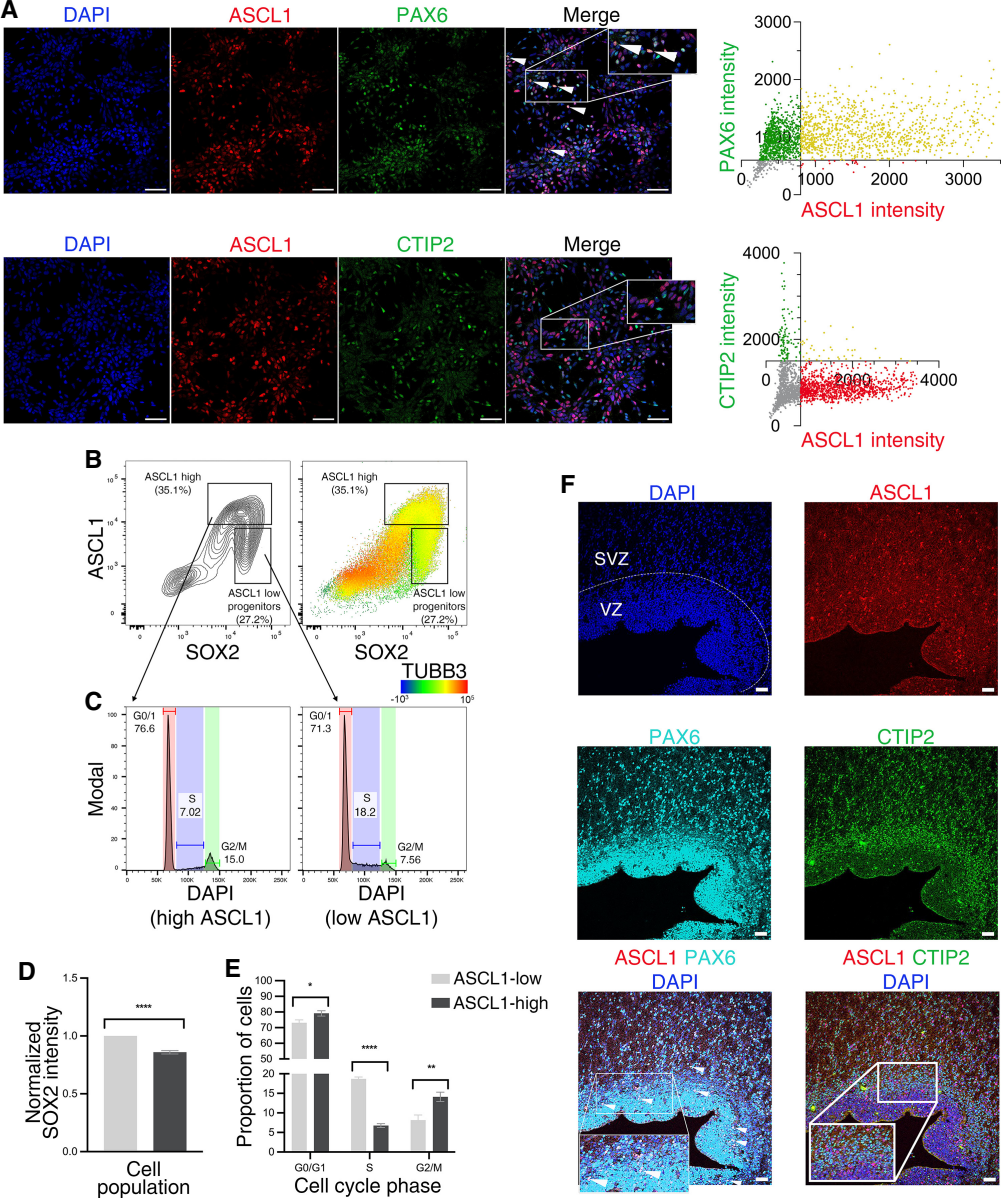
ASCL1 protein expression also marks a transitional progenitor population poised to differentiate. (*A*) Immunofluorescence images of DIV24 neural cultures showing cells colabeled for ASCL1 and the progenitor marker PAX6 (*top*; e.g., white arrowheads), but not for ASCL1 and the neuronal marker CTIP2 (BCL11B; *bottom*). Scale bar, 50 μm. (*Right*) Quantification of PAX versus ASCL1 (*top*) and CTIP2 versus ASCL1 (*bottom*) nuclear immunofluorescence intensity is shown. Yellow indicates coexpression: 99.8% of ASCL1^+^ coexpress PAX6, and 3.6% coexpress CTIP2. (*B*) Flow cytometry profiles showing a contour plot of intensities of labeling for ASCL1 and SOX2 in single cells analyzed at DIV24 (*left*) and a dot plot equivalent to the contour plot at the *left*, pseudocolored according to the level of TUBB3 expression (*right*; key shown at the *bottom right*). *Insets* indicate ASCL1-high and ASCL1-low populations, respectively. (*C*) DNA content histogram profiles obtained by flow cytometry after DAPI staining for the ASCL1-high (*left*) and ASCL1-low (*right*) progenitor populations defined in *B*, indicating the cell cycle distribution of cells in these populations. (*D*) Quantification of normalized SOX2 intensities in ASCL1-high and ASCL1-low populations from *B*. Error bars represent mean ± SEM for three biological replicates. Unpaired *t*-test: (****) *P* < 0.0001. (*E*) Quantification of the proportion of cells in different cell cycle phases as a percentage of the total cell population (cell cycle analysis by flow cytometry, as analyzed in *C*), showing significant accumulation of cells in S phase in the ASCL1-low population and accumulation of cells in G1 and G2/M in the ASCL1-high population. Error bars represent mean ± SEM for three biological replicates. Unpaired *t*-test: (*) *P* < 0.05, (**) *P* < 0.01, (****) *P* < 0.0001. (*F*) PCW 16 fetal brain slice coimmunolabeled for ASCL1, PAX6, and CTIP2, showing colabeling of ASCL1 with progenitor marker PAX6 (e.g., white arrowheads) but not with deep-layer marker CTIP2 (BCL11B). Scale bar, 50 μm. (VZ) Ventricular zone, (SVZ) subventricular zone.

We next sought to further characterize this unique cluster of *ASCL1*-expressing progenitors by investigating coexpression of the ASCL1 protein with canonical markers of cell identity. Using immunolabeling of DIV24 cultures, we found that ASCL1 was expressed in the granular “salt-and-pepper” pattern characteristic of proneural factors (Kageyama et al. 2008). While all ASCL1-positive cells coexpressed the neural progenitor marker PAX6, none coexpressed the deep-layer cortical neuronal marker CTIP2, confirming that ASCL1 is expressed by a subset of progenitor cells and not by differentiated neurons (Fig. 2A). Flow cytometry analysis of dissociated DIV24 cultures confirmed this with coexpression of ASCL1 and the progenitor marker SOX2 but not with the neuronal marker TUBB3 (Fig. 2B). Interestingly, ASCL1 immunolabeling signal was highest in cells bridging progenitors (low TUBB3 and high SOX2) and neurons (high TUBB3 and low SOX2), indicating that the ASCL1 protein is expressed at its highest levels before the reduction of SOX2 expression and the up-regulation of TUBB3 expression (Fig. 2B,D), similar to the distribution of *ASCL1* transcripts in transitional progenitors in the scRNA-seq analysis (Fig. 1E). Analysis of nuclear DNA content to infer cell cycle stage showed a significantly lower proportion of cells in S phase in ASCL1-high compared with ASCL1-low progenitors (Fig. 2C,E). This was accompanied by greater proportions of cells in G0/G1 and G2/M phases in the ASCL1-high population (Fig. 2C,E). Taken together, these data suggest that a large proportion of cells from the ASCL1-high population progress through their last division and exit the cell cycle, presumably before differentiating into neurons.

To confirm the relevance of these findings in vivo, we investigated ASCL1 expression in the developing human cortex using human fetal brain tissue at postconceptional week 16 (PCW 16). Immunolabeling of human fetal brain slices for ASCL1 and PAX6 or CTIP2 showed that during human cortical development, ASCL1 expression is also restricted to PAX6-positive progenitors and excluded from postmitotic neurons (Fig. 2F), as previously reported (Alzu'bi and Clowry 2019), corroborating the findings in our in vitro model system of human neurodifferentiation.

### ASCL1 drives the differentiation of cycling progenitors into postmitotic neurons by directly regulating hundreds of genes

**Figure 3.**
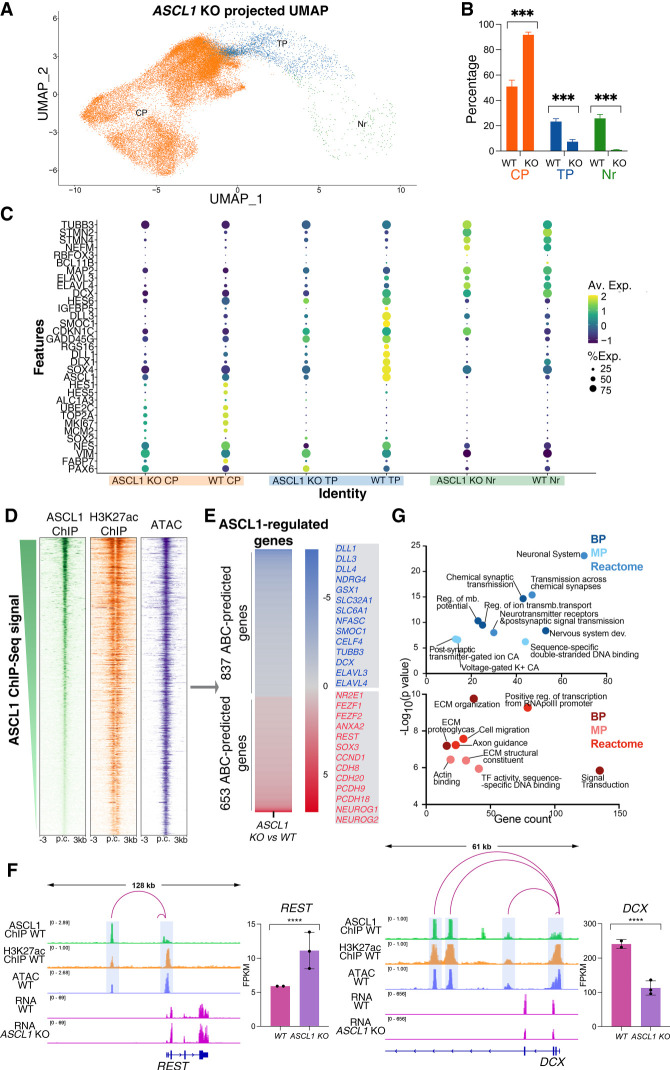
ASCL1 binds to distal regulatory elements of many target genes involved in neural development. (*A*) Single-cell transcriptomes from 35,755 differentiating *ASCL1* KO neural cells at DIV24, collected from three independent cultures, projected onto the wild-type cells UMAP embedding represented in Figure 1D. (*B*) Relative proportion of major cell clusters from *A* in comparison with the cell clusters in Figure 1D. Loss of *ASCL1* results in significantly reduced proportions of transitional progenitors (TPs) and neurons (Nrs) and a significant increase in cycling progenitors (CPs). Unpaired *t*-test: (***) *P* < 0.001. (*C*) Dot plot representation of the expression of biologically relevant genes in the major clusters from Figure 1D, showing expression differences between WT and *ASCL1* KO cells mapped to those clusters. Dot size indicates proportion of cells in cluster expressing a gene, and shading indicates the relative level of expression. (*D*) Heat maps representing ChIP-seq coverage for ASCL1 binding sites at genomic regions predicted to be regulatory elements by the ABC algorithm (based on their chromatin accessibility and H3K27ac signature) (Fulco et al. 2019). (p.c.) Peak center. (*E*) ASCL1-regulated genes; i.e., genes selected after enhancer–gene regulatory relationship was predicted with the ABC algorithm and where enhancers were identified to be bound by ASCL1 via ChIP-seq, and subsequently the regulated genes were found to be differentially expressed in bulk RNA-seq analysis of *ASCL1* KO versus wild-type cells (Supplemental Fig. S2). Color coding indicates differential gene expression fold change in *ASCL1* KO versus wild-type cultures. Illustrative genes for each category are highlighted (listed in Supplemental Table S1). (*F*) Representative Integrative Genomic Viewer (IGV) tracks of ChIP-seq, ATAC-seq, and bulk RNA-seq data illustrating examples of ASCL1-occupied active regulatory elements (as predicted by ABC algorithm) targeting genes up-regulated (*left*) or down-regulated (*right*) in *ASCL1* KO versus control cultures at DIV24. Bar plots show mean expression in FPKM for the depicted genes in wild-type and *ASCL1* KO cultures. (****) *P*-adj < 0.0001. (*G*) Graphical representation of log transformed *P*-value and gene number for top enriched GO biological process terms ([BP] biological process, [MP] molecular process) and reactome pathways for the genes down-regulated (*top*; blue) and up-regulated (*bottom*; red) in *ASCL1* KO cells at DIV24, as displayed in *E* (complete list in Supplemetal File S3).

Having defined a unique ASCL1-expressing population consisting of progenitor cells exiting the cell cycle, we sought to investigate the role of ASCL1 in that transition. To that end, we generated *ASCL1* knockout (*ASCL1* KO) cells by using CRISPR/Cas9 to create a frameshift-inducing deletion in the same iPSC line (Supplemental Fig. S2A). We genomically screened three independent clones for frameshift mutations, differentiated them to DIV24, and performed ASCL1 detection by Western blotting. All mutant clones showed an absence of ASCL1 protein even at high chemiluminescent exposure (Supplemental Fig. S2B), thus confirming the generation of *ASCL1*-null mutant cells. To address *ASCL1* function in iPSC-derived differentiating neural cultures, we performed scRNA-seq on DIV24 cultures of the three *ASCL1* KO clones (in parallel with the wild-type clones shown in Fig. 1), yielding 35,755 single cells. When we projected this new single-cell gene expression data set onto the reference wild-type UMAP embedding from Figure 1D, we found an increase in the number of cycling progenitors in mutant cells compared with wild-type controls, accompanied by a dramatic reduction in the proportions of transitional progenitors and a near absence of neurons (Fig. 3A,B). Moreover, the small number of mutant cells assigned to the transitional progenitors had a different transcriptional signature when compared with control cells (Fig. 3C). We also independently integrated scRNA-seq data sets for WT and *ASCL1* KO transcriptomes and assigned cluster identity based on the previously defined markers (Supplemental Fig. S2C). This analysis corroborated the previous finding of transitional progenitor and neuron depletion in the *ASCL1* KO data set (Supplemental Fig. S2C,D). When *ASCL1* KO and wild-type cells are integrated, the de novo UMAP representation displays poor clustering of *ASCL1* KO cells with WT cells in the transitional progenitor and neuron clusters, also supporting that the overall transcriptional signatures of the two genotypes are different in these two cell types (Supplemental Fig. S2E). Together, these data suggest that loss of *ASCL1* impedes cell cycle exit and differentiation even in the presence of a potent NOTCH inhibitor (DAPT), which was insufficient to induce cell cycle exit in the absence of ASCL1, resulting in an accumulation of cells in a proliferating and undifferentiated state.

Because overexpression of ASCL1 has been shown to drive changes in cell fate through its pioneer transcription factor activity (Wapinski et al. 2013, 2017; Chanda et al. 2014; Raposo et al. 2015), we hypothesized the same function could underlie its endogenous role in human neurogenesis. Therefore, we investigated *ASCL1* function in transcriptional regulation at DIV24. First, we analyzed transcriptional changes by bulk RNA sequencing of DIV24 cultures in *ASCL1* KO versus control cells. We opted to perform RNA-seq in independent bulk cultures to ensure concurrently cultured wild-type and *ASCL1* KO cells were available for parallel molecular experiments described below, currently not amenable to single-cell techniques. We identified 2562 differentially expressed genes (fold change > 1.5, *q*-value < 0.05), including 1451 down-regulated and 1111 up-regulated genes in KO cells (Supplemental Fig. S2F; Supplemental File S1). We next sought to determine whether these transcriptional changes were due to functional binding activity of ASCL1.

First, we predicted the genome-wide map of enhancer–promoter interactions specific to our model system (Fulco et al. 2019) using the activity by contact (ABC) computational algorithm. For this, we characterized the chromatin accessibility landscape by accessible chromatin with sequencing (ATAC-seq) and the genomic profile of the H3K27ac histone mark, which correlates with active enhancers (Creyghton et al. 2010), by ChIP-seq in wild-type DIV24 cultures. Integrating the cell type-specific expression from our wild-type DIV24 RNA-seq data set, we were able to predict the enhancer–gene regulatory connections in our human differentiating neural culture system. We identified a repertoire of 23,662 active regulatory elements associated with 10,430 expressed genes (with each gene regulated by one or more elements) (Supplemental File S2).

We then characterized the genomic binding profile of ASCL1 by chromatin immunoprecipitation coupled with high-throughput sequencing (ChIP-seq). Analysis of ASCL1 ChIP-seq at DIV24 revealed 56,100 significant binding sites, with 68.3% found in ATAC-accessible chromatin, of which 75.9% were characterized by H3K27ac histone marks. The majority of ASCL1-bound sites (70.3%) were intergenic or located in gene introns. The average distance between ASCL1 binding targets and the nearest transcription start sites (TSSs) was 33.3 kb, with most (82.5%) locating >1 kb from a TSS. Based on the above ABC-predicted regulatory landscape, ASCL1 bound 17,049 active enhancer regions in WT DIV24 (Fig. 3D). These data are consistent with ASCL1 binding regulating neurogenesis by predominantly binding at distal regulatory elements (DRE). Having thus predicted the regulatory landscape in wild-type cells, we sought to characterize the role of ASCL1 by investigating the effect of *ASCL1* depletion. From the 2562 genes found to be differentially expressed in *ASCL1* KO cells (Supplemental Fig. S2F), ASCL1 binds at least one of the ABC-predicted regulatory elements assigned to 58.5% (1490) of these (Fig. 3E). This repertoire of 1490 genes, referred to here as “putative ASCL1-regulated genes,” at DIV24 includes known markers of neuronal differentiation; e.g., neuronal genes *TUBB3* and *DCX* (down-regulated in *ASCL1* KO cells) and neuronal transcriptional repressor *REST* (up-regulated in *ASCL1* KO cells) (Fig. 3F; Supplemental File S3; Ballas et al. 2005). Focusing our analysis on ASCL1-regulated genes ensured our analysis was not biased by indirect gene expression changes downstream from ASCL1 or reflecting differences in cell type composition of the cultures. Gene ontology analysis of the down-regulated *ASCL1* target genes revealed an enrichment of biological processes linked to neuronal differentiation and neuronal activity. Conversely, GO terms associated with up-regulated *ASCL1* target genes highlighted processes prominent in extracellular matrix, cell migration, and axon guidance (Fig. 3G; Supplemental File S3), consistent with previous evidence for ECM gene repression in direct conversion of fibroblasts to neurons by ASCL1 (Wapinski et al. 2017). These experiments therefore establish *ASCL1* as a direct *cis*-regulator of neural loci during human neurogenesis.

### ASCL1 has pioneer transcription factor activity at a subset of its targets during human neurogenesis

**Figure 4.**
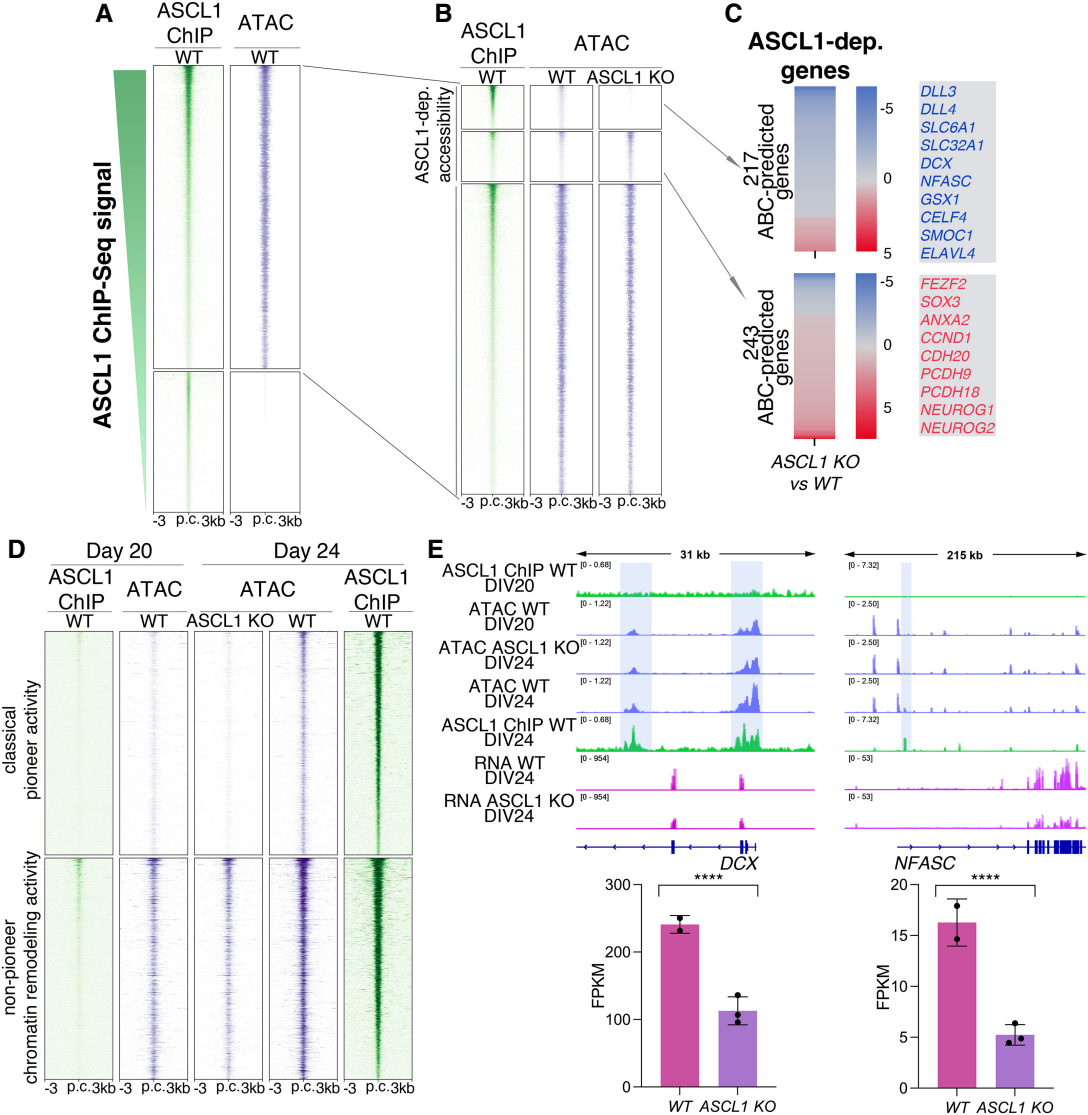
ASCL1 regulates chromatin accessibility through different pioneer and nonpioneer functions. (*A*) Heat maps showing ASCL1 binding sites identified by ChIP-seq in open (*top*; *n* = 38,377) and closed (*bottom*; *n* = 17,723) chromatin, as determined by ATAC-seq in DIV24 neural cultures. (*B*) Heat maps showing open chromatin sites in DIV24 (from *A*) where ASCL1 regulates accessibility (i.e., “ASCL1-dependent” sites) by opening (*top*; *n* = 4288) or closing (*middle*; *n* = 4896) chromatin, and where ASCL1 binds without regulating accessibility (*bottom*; *n* = 29,220). (*C*) Heat maps showing the differential gene expression for ABC-predicted genes associated with regulatory elements at which ASCL1 promotes (*top*) or represses (*bottom*) chromatin accessibility (from *B*), and dysregulated in *ASCL1* KO versus wild-type DIV24 cultures. Decreased accessibility is associated with decreased expression in 79.3% of ASCL1-regulated genes; conversely, 74.9% of ASCL1-regulated genes showed increased accessibility of regulatory elements associated with increased gene expression. Color coding indicates differential gene expression fold change versus wild-type culture. Illustrative genes for each category are highlighted at the *right*. (*D*) Heat maps showing the changes in chromatin accessibility between wild-type DIV24 cultures (*right*) and wild-type DIV20 cultures (*left*) and ASCL1 mutant DIV24 cultures (*middle*) at ASCL1-dependent sites (from *B*) where ASCL1 opens chromatin, acting as a classical pioneer transcription factor (*top*, *n* = 2155), and where its activity changes accessibility at permissive sites (*bottom*, *n* = 2133). (*E*, *top*) Representative IGV tracks flanking a putative ASCL1-dependent gene showing ChIP-seq and ATAC-seq profiles at DIV20 and DIV24 (from *D*). (*Bottom*) Bar plots show mean expression in FPKM for the depicted genes in wild-type and *ASCL1* KO cultures. (****) *P*-adj < 0.0001.

We next investigated the functional relationship between ASCL1 binding and changes in chromatin state. The pioneer transcription factor function of ASCL1 has been established, with evidence for the ability of the overexpressed protein to bind closed chromatin and promote local DNA accessibility, allowing the binding of factors that do not share such a pioneer activity and regulating cell fate (Wapinski et al. 2013; Chanda et al. 2014; Raposo et al. 2015; Park et al. 2017; Aydin et al. 2019). However, the pioneer activity of the endogenous protein has not been explored. We therefore investigated whether the regulation of the identified putative *ASCL1*-regulated genes represented canonical features of pioneer transcription factor activity, with a functional link between binding of ASCL1 and chromatin accessibility. We first examined how ASCL1 binding correlated with chromatin state in DIV24 cultures by overlapping the wild-type ASCL1 ChIP-seq and ATAC-seq data sets. We found that 31.7% of the 56,100 ASCL1-bound sites in DIV24 cultures were in closed chromatin and 68.3% were in accessible chromatin in the ATAC-seq data set, revealing a strong correlation between ASCL1 binding and accessible chromatin at the time point coincident with high ASCL1 expression (Fig. 4A). Next, to investigate whether ASCL1 is involved in regulating chromatin structure, we examined chromatin accessibility in DIV24 *ASCL1* KO cultures by ATAC-seq and compared these with the ATAC-seq data sets from DIV24 WT. Among the ASCL1-bound sites in open chromatin in wild-type cells (Fig. 3D), 9156 (23.9%) showed changes in accessibility; 46.8% of these showed decreased accessibility in the *ASCL1* KO, and 53.2% regions showed increased accessibility (Fig. 4B). Using our predetermined ABC-predicted gene-regulatory element connections (Fig. 3D), we assigned 460 *ASCL1*-regulated genes to the loci of differential accessibility in *ASCL1* KO versus wild-type cells. Differential regulatory element accessibility was largely consistent with differential gene expression in the *ASCL1* KO (Fig. 4B,C). These results agree with the notion that pioneer TFs can promote both activation and repression of gene expression depending on secondary factors accessing the opened chromatin (Hosokawa et al. 2018; Zaret 2020). Our analysis therefore supports that *ASCL1* controls expression of a fraction of its targets by regulating the chromatin accessibility landscape at active regulatory elements, referred to here as putative “*ASCL1*-dependent” genes, thus acting as a pioneer TF.

To gather further evidence for ASCL1 pioneer activity at these ASCL1-dependent sites, we examined the temporal dynamics of chromatin accessibility in differentiating neural cultures and its relationship with ASCL1 binding. We compared chromatin accessibility in wild-type cells at DIV24, when ASCL1 expression is at its peak, with wild-type cultures 4 d earlier, at DIV20, when ASCL1 expression is still very low (Fig. 1A,B). Because ATAC-seq is a population-wide genomic assay and likely to reflect both direct and indirect effects of ASCL1 transcriptional activity, we focused our analysis on sites bound by ASCL1 in wild-type DIV24 cultures. We observed that the DIV24 *ASCL1* KO accessibility profile mirrored the DIV20 prebound state (Fig. 4D,E); 74.7% of sites differentially closed at DIV20 versus wild-type DIV24 were also closed in *ASCL1* KO DIV24 cells.

We then observed two categories of sites that lost accessibility in *ASCL1* KO cultures; 50.3% exhibited features of interaction with a classical pioneer factor—i.e., open in wild-type cells and completely closed in *ASCL1* KO cells (and similarly in DIV20 progenitors) (Fig. 4D, top). The remaining 49.7% of sites showed a significant decrease in accessibility (fold change > 1.5, *q*-value < 0.05) but were not devoid of ATAC signal in *ASCL1* KO cultures, contrary to the first category; i.e., chromatin was permissive in the absence of ASCL1 (Fig. 4D, bottom). This shows that ASCL1 pioneer activity is not required at those sites, but its presence increases chromatin accessibility, suggesting that another factor is involved in opening chromatin at those sites and that ASCL1 may act as a partner to further remodel chromatin (Henke et al. 2009; Fernandez Garcia et al. 2019). To explore this possibility, we investigated whether motif content distinguished sites where ASCL1 acts as a classical pioneer and opens chromatin from those where it binds at permissive DNA and is required for further chromatin remodeling. In both categories, the top enriched motif discovered was a bHLH binding motif with the motif core containing the previously reported 5′-CAGCTG-3′ ASCL1 consensus binding sequence (Supplemental File S4; Raposo et al. 2015). This lack of significant difference in motif enrichment suggests that a non-sequence-specific DNA binding factor (e.g., a chromatin remodeling complex) may be responsible for opening chromatin at those sites where ASCL1 is found to bind permissive DNA. We identified similar dynamics for the sites where ASCL1 represses chromatin accessibility, though here we found evidence for classical pioneer activity at only 15.6% of sites that are closed versus open in wild-type DIV24 versus *ASCL1* KO cultures (Supplemental Fig. S4A, top). This is in keeping with a less prominent role of ASCL1 as a repressive pioneer factor than reported for other pioneer factors (Wapinski et al. 2017; Hosokawa et al. 2018; Zaret 2020). Overall, 23.5% of all ASCL1-dependent sites are subject to its “classical” pioneer activity in human neural cultures (i.e., binding to closed chromatin and remodeling state), while 68.2% of ASCL1 binding sites are consistent with chromatin remodeling at sites of low accessibility in the ASCL1 naïve state (i.e., with a nonpioneer chromatin remodeling activity at those sites) (Fig. 4D; Supplemental Fig. S4A, bottom).

### ASCL1 interacts with mSWI/SNF chromatin remodelers at sites where it acts as a pioneer factor

Given the evidence that ASCL1 regulates chromatin accessibility (with or without pioneer activity) in differentiating human neural cultures, we hypothesized that ASCL1 may regulate chromatin states by interacting with chromatin remodelers and specifically with ATPase-dependent chromatin remodeling complexes (Zaret and Carroll 2011). mSWI/SNF or BRG1/BRM-associated factor (BAF) complexes are evolutionarily conserved ATP-dependent chromatin remodeling complexes with pivotal roles in neural development, in particular at the transition from neural progenitors to neurons (Lessard et al. 2007; Yoo et al. 2009; Braun et al. 2021). Because our above data support ASCL1 peak expression coinciding with a progenitor state poised to differentiate, we hypothesized that ASCL1 functionally interacts with mSWI/SNF complexes at this crucial stage of neurogenesis.

In order to test this hypothesis, we first examined the expression patterns of mSWI/SNF subunits and compared them with the ASCL1 expression pattern in our in vitro model system of neuronal differentiation (Fig. 1A,B). Core subunits are expressed at all stages of neural differentiation in these cultures (Supplemental Fig. S3A). Supporting the previously reported npBAF–nBAF subunit switch in mouse neurogenesis, we observed a decrease in the expression of progenitor-specific subunits ACTL6A, SS18, and DPF1/DPF3, accompanied by an increase in the expression of neuronal-specific subunits ACTL6B, SS18L1, and DPF2/PHF10, as cells differentiated from neural progenitors into postmitotic neurons (Supplemental Fig. S3B). Consistent with our findings of impaired generation of transitional progenitors and neurons in *ASCL1* KO cultures, we also observed in the bulk RNA-seq data set a decreased expression of all nBAF-specific subunits in the *ASCL1* KO DIV24 cultures but no change in the npBAF subunits (Supplemental Fig. S3C). In addition, ASCL1 was expressed in cells containing the npBAF subunit ACTL6A but not the nBAF-specific subunit ACTL6B, consistent with ASCL1 being expressed in neural progenitor cells and not in postmitotic neurons (Supplemental Fig. S3A,D).

**Figure 5.**
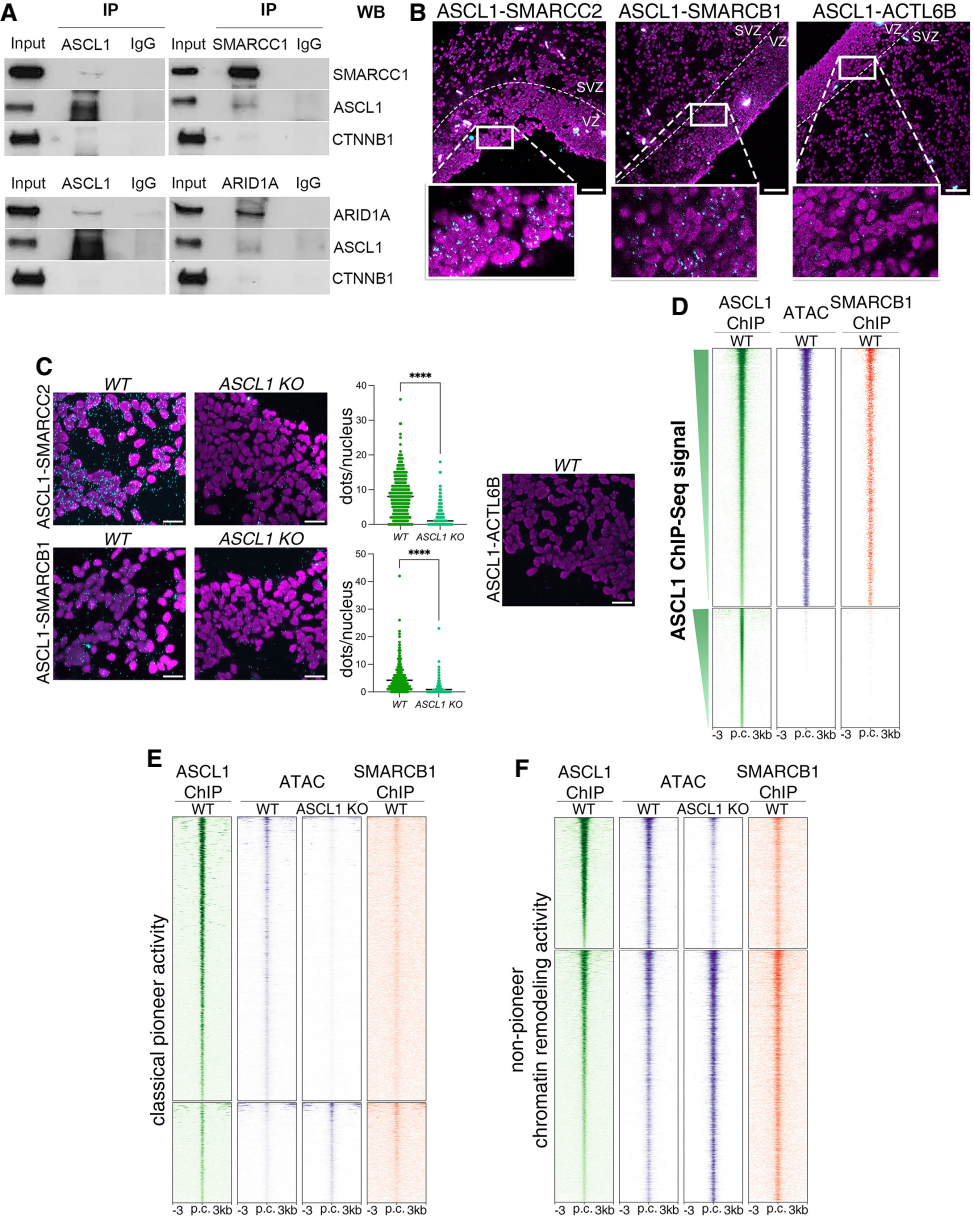
ASCL1 interacts with mSWI/SNF remodeling complexes predominantly at sites where it does not have classical pioneer activity. (*A*) Immunoprecipitation followed by Western blot analysis in DIV24 neural cultures showing reciprocal coimmunoprecipitation of ASCL1 and SMARCC1 or ARID1A. CTNNB1 loading control is included. (*B*,*C*) Representative immunofluorescence images of proximity ligation assay between ASCL1 and SMARCC2, SMARCB1, or ACTL6B in the human fetal cortex at PCW 16 (*B*) and in wild-type and *ASCL1* KO DIV24 neural cultures (*C*). Cyan foci indicate PLA amplification signal. Nuclei are shown in magenta (DAPI). (*C*) Numbers of foci per nucleus were quantified in three nonoverlapping fields of view. Scale bars, 50 μm. The no antibody control is shown in Supplemental Figure S3E. Unpaired *t*-test: (****) *P* < 0.0001. (*D*) Heat maps of SMARCB1 binding from ChIP-seq analysis (*right*) at all ASCL1 binding sites (*left*; from Fig. 4A, redisplayed for comparison) found in open (*top*; *n* = 38,377) or closed (*bottom*; *n* = 17,723) chromatin (*middle*) in DIV24 neural cultures. Sites cobound by ASCL1 and SMARCB1 are mostly found in regions of open chromatin. (*E*,*F*) Heat map profiles of SMARCB1 binding (*right*) in DIV24 neural cultures at ASCL1-dependent sites (*left*; from Fig. 4, redisplayed for comparison) where ASCL1 acts as a classical pioneer factor binding closed chromatin (ATAC signal below threshold in *ASCL1* KO cultures; *E*, *middle right*) and sites where ASCL1 binds permissive chromatin (nonpioneer chromatin remodeler; ATAC signal shows open signal in *ASCL1* KO cultures, but ASCL1 causes changes in accessibility; *F*, *middle right* vs. *middle left*). (*F*, *top* and *bottom*) SMARCB1 is enriched at sites where ASCL1 binds open chromatin and changes accessibility.

Based on the observation that ASCL1-expressing cells (predominantly transitional progenitors) coexpress npBAF subunits and our hypothesis above that ASCL1 and BAF functionally interact, we then investigated whether that coexpression reflected a physical interaction between the two. We first performed immunoprecipitation of ASCL1 from a lysate of DIV24 neural cultures followed by Western blot analysis (co-IP) and found that ASCL1 coimmunoprecipitates with mSWI/SNF subunits SMARCC1 and ARID1A; reciprocally, immunoprecipitates of SMARCC1 and ARID1A also contained ASCL1 (Fig. 5A). We then examined the interaction in situ by proximity ligation assay (PLA) conducted in DIV24 control and *ASCL1* KO neural cultures (Fig. 5B) and on slices of PCW 16 human fetal cortex (Fig. 5C). PLA analysis was conducted with antibodies for ASCL1 and for the mSWI/SNF core subunits SMARCC2 and SMARCB1 as well as the neuronal subunit ACTL6B, and PLA signal was detected for both ASCL1–SMARCC2 and ASCL1–SMARCB1 pairs, but not for the ASCL1–ACTL6B-negative control pair, in both our in vitro model system and ex vivo human tissue (Fig. 5B,C). Together, these results indicate that ASCL1 physically interacts with mSWI/SNF chromatin remodeling complexes during neural development in vitro and in vivo.

To investigate whether this physical interaction between ASCL1 and mSWI/SNF complexes is relevant for coregulation of epigenetic states, we first determined the genome-wide binding profile of the mSWI/SNF core subunit SMARCB1 in DIV24 neural cultures by ChIP-seq. We found that 90.3% of SMARCB1 binding sites overlapped with nucleosome-depleted regions previously identified by ATAC-seq (Supplemental Fig. S4C), consistent with its role in chromatin remodeling (Bao et al. 2015; Schick et al. 2019; Iurlaro et al. 2021). Assuming that the physical interaction between ASCL1 and mSWI/SNF is involved in chromatin regulation, we then overlapped ASCL1 (Fig. 4A) and SMARCB1 binding sites; 54.9% of the ASCL1 targets in open configuration at DIV24 also displayed SMARCB1 binding, of which 85.5% were characterized by H3K27ac marks, while only 5.8% of ASCL1 targets in closed configuration in DIV24 were cobound by SMARCB1, indicating that corecruitment relates to accessible chromatin (Fig. 5D). In addition, motif analysis revealed that the motif enriched at the largest number of targets (52.2%) includes the 5′-CAGCTG-3′ ASCL1 consensus binding sequence at its core (Raposo et al. 2015; Supplemental File S4C), suggesting that the sequence binding specificity of the cooperative pair is conferred by ASCL1. Conversely, the ASCL1 motif is not identified in sites bound only by SMARCB1 (Supplemental File S4D).

Because we had identified two classes of ASCL1-dependent loci—one where ASCL1 classical pioneer activity is required and one where ASCL1 acts at a nonpioneer remodeling factor, changing chromatin state on already permissive DNA, respectively (Fig. 4D)—we hypothesized that the requirement of ATP-dependent cofactors might differ between the two classes. Indeed, at sites where ASCL1 acts as a classical pioneer factor, we identified SMARCB1 ChIP-seq cobinding at only 12.7% of them (Fig. 5E). Conversely, at sites where ASCL1 affects accessibility without the classical features of a pioneer factor, 53.1% of them are cobound by SMARCB1 (Fig. 5F). Moreover, examining the SMARCB1 ChIP-seq data, we found that at most of the remaining 46.9% of sites, there is some SMARCB1 ChIP-seq signal that is below threshold for peak calling. With these data, we conclude that ASCL1 and mSWI/SNF interact at sites where ASCL1 is involved in chromatin remodeling, and that this interaction is more predominant at sites where ASCL1 lacks classical pioneer activity.

### ASCL1 works in concert with the mSWI/SNF ATPase complexes to remodel chromatin and regulate gene expression during human neurogenesis

Given that mSWI/SNF is enriched at ASCL1-dependent sites, particularly at sites where ASCL1 is not required to open chromatin but acts to increase accessibility, we hypothesized that ASCL1 requires mSWI/SNF complexes to regulate chromatin accessibility at those sites. To address this, we investigated the effect of suppressing mSWI/SNF activity on ASCL1 function. We first chose to acutely eliminate the mSWI/SNF complexes by simultaneous CRISPR/Cas9-mediated knockout of the *SMARCC1* and *SMARCC2* core subunits in DIV21 cultures (preceding the ASCL1 expression peak), with analysis of the mutant cultures 3 d later at DIV24. Elimination of both core subunits (Supplemental Fig. S4D) resulted in the depletion of other mSWI/SNF subunits, likely reflecting destabilization of the entire mSWI/SNF complex in the absence of its core subunits (Supplemental Fig. S4E), in agreement with reports in other models (Narayanan et al. 2015; Mashtalir et al. 2018; Schick et al. 2019). Importantly, in addition to poor cell viability with almost complete depletion of mSWI/SNF complexes, ASCL1 expression itself was down-regulated in SMARCC1/C2 mutant cells (Supplemental Fig. S3F), limiting the value of this model for investigating the role of mSWI/SNF–ASCL1 interaction.

We thus chose the alternative approach of using the small molecule BRM014 to inhibit the catalytic activity of the two ATPases of mSWI/SNF complexes: SMARCA2 and SMARCA4 (Papillon et al. 2018; Iurlaro et al. 2021). We exposed DIV22 neural cultures to 10 mM BRM014 and harvested the cultures for analysis 48 h later, at DIV24 (1 d after NOTCH inhibition, at ASCL1 expression peak). ASCL1 expression was not affected by exposure to BRM014 (Supplemental Fig. S4G). We first investigated how BRM014 treatment affected mSWI/SNF complex enzymatic activity by examining its effect on chromatin accessibility using ATAC-seq in BRM014-treated and control cells. We found that BRM014-treated cells presented reduced chromatin accessibility at 3664 genomic sites and increased accessibility at 277 sites (*q*-value < 0.5), with 68.6% of the changes in accessibility occurring at SMARCB1-bound sites. These results were in keeping with the anticipated effects of inhibiting mSWI/SNF enzymatic activity without compromising ASCL1 expression, indicating that this is a suitable system to investigate mSWI/SNF–ASCL1 interactions.

**Figure 6.**
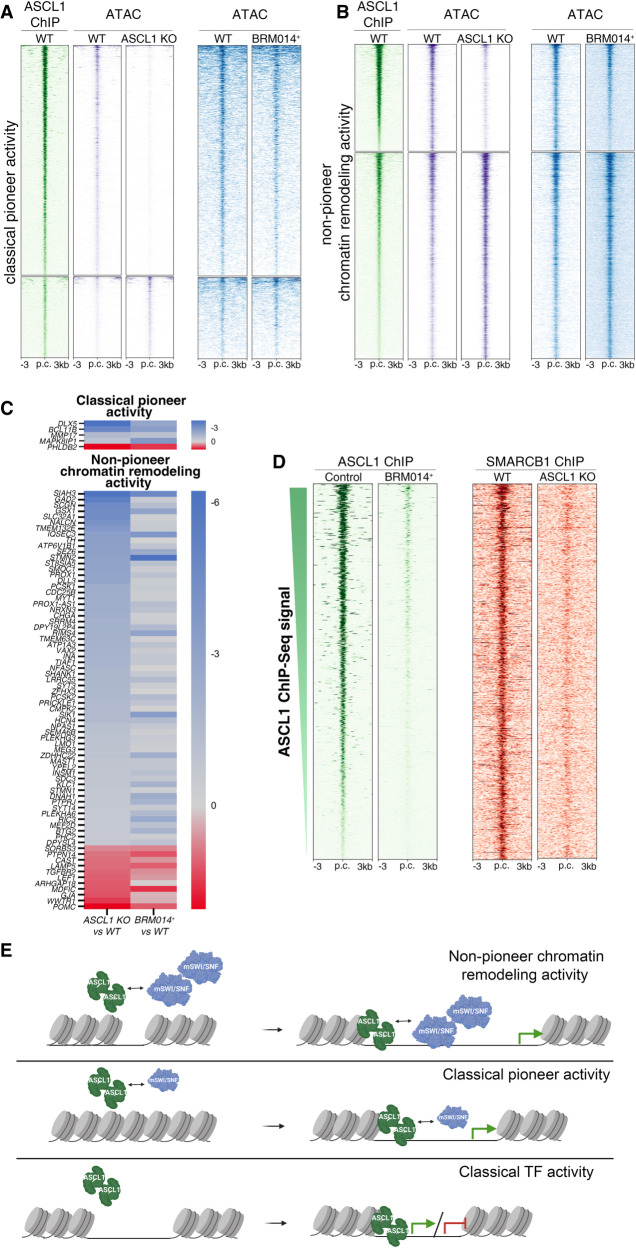
ASCL1 works in concert with mSWI/SNF complexes to remodel chromatin and regulate gene expression. (*A*,*B*) Heat maps showing the effect of BRM014 treatment on chromatin accessibility at ASCL1-bound sites where ASCL1 displays classical pioneer activity (*A*) or nonpioneer chromatin remodeling activity (*B*). (*B*) The ATPase activity of mSWI/SNF complexes is mainly required at sites of nonpioneer activity. ASCL1 ChIP-seq (Fig. 5E) is included for reference. (*C*, *top*) Heat map of differential gene expression for genes dysregulated in both *ASCL1* KO and BRM014-treated cultures and whose ABC-predicted regulatory element(s) are associated with ASCL1–SMARCB1-cobound sites where both are required to increase accessibility and where ASCL1 acts as a classical pioneer factor. (*Bottom*) Heat map of differential gene expression for genes dysregulated in both *ASCL1* KO and BRM014-treated cultures and whose ABC-predicted regulatory element(s) are associated with ASCL1–SMARCB1-cobound sites where both are required to increase accessibility and where ASCL1 acts as a nonpioneer chromatin remodeler. (*D*) Heat maps profiling ASCL1 and SMARCB1 binding at ASCL1–mSWI/SNF-dependent sites where the interaction is associated with open chromatin, showing that interfering with mSWI/SNF ATPase activity (BRM014 treatment) reduces ASCL1 binding and, reciprocally, that eliminating ASCL1 (*ASCL1* KO) reduces SMARCB1 binding. (*E*) Diagram illustrating the proposed mechanisms for the ASCL1 transcription factor activity and ASCL1–mSWI/SNF recruitment dynamic. (*Top*) ASCL1 has nonpioneer chromatin remodeling activity, binding cooperatively with mSWI/SNF at sites of accessible chromatin. (*Middle*) ASCL1 has pioneer activity, binding cooperatively with mSWI/SNF at inaccessible regulatory elements, where they regulate accessibility. (*Bottom*) ASCL1 acts as a classical transcription factor (TF), binding accessible regulatory elements to regulate transcription of target genes. (Created with BioRender.com.)

To address whether ASCL1 requires mSWI/SNF to regulate chromatin states, we focused our attention on ASCL1 sites where ASCL1 binding significantly affects chromatin accessibility (see Fig. 4). When we plotted the overall accessibility changes at these ASCL1-dependent sites in *ASCL1* KO cells and BRM014-treated cells (without BRM014-treated *q*-value or fold change threshold), we found a positive correlation (*r* = 0.395) between the two conditions (Fig. 6A,B; Supplemental Fig. S4H). We found a similar correlation when comparing the transcriptional effects of both conditions (Supplemental Fig. S4I), indicating that the interaction of ASCL1 with mSWI/SNF is relevant to downstream transcriptional activity. Hence, we reasoned that the ASCL1–mSWI/SNF interaction inducing changes to chromatin structure in transitional progenitors would have relevance to the regulation of neural loci. We identified 2408 sites that are cobound by ASCL1 and SMARCB1 and where the direction in change in accessibility is concordant in the two conditions (*ASCL1* KO at *q* < 0.5, and BRM1-treated threshold-free). These sites corresponded to 1259 ABC-predicted regulatory elements (Fulco et al. 2019) that control the expression of 141 genes during human neurogenesis, corresponding to 30.7% of the putative *ASCL1*-dependent genes and including important genes involved in neural development; e.g., *DLL3*, *MYT1*, *STMN1*, and *SHANK1* activated by both partners (Fig. 6C).

Importantly, for the 3315 ASCL1-dependent sites where ASCL1 binds permissive DNA and is cobound by SMARCB1 (Fig. 5F), 53.1% are coregulated by mSWI/SNF activity, corresponding to 1195 predicted regulatory elements assigned to 132 genes. Conversely, we only identified 370 ASCL1-dependent sites where ASCL1 acts as a classical pioneer TF and is cobound by SMARCB1, with codependency for 45.1%, corresponding to 64 predicted regulatory elements assigned to nine genes. These results indicate that functional interaction of ASCL1 and ATPase-active mSWI/SNF is predominantly at sites where chromatin is already partially or transiently opened.

Having identified cobinding by ASCL1 and mSWI/SNF and coregulation of chromatin accessibility, we then asked whether chromatin remodeling activity of one factor at the cobound sites is required for binding of the other, or whether each factor can bind independently of the other. To investigate this, we performed reciprocal SMARCB1 and ASCL1 ChIP-seq in *ASCL1* KO and BRM014-treated cells, respectively. At 92.7% of these putatively coregulated sites, we found evidence for a significant decrease or complete absence of DNA binding of the partner (Fig. 6D). Residual binding of SMARCB1 is consistent with the limited chromatin accessibility observed in *ASCL1* KO cells at sites of nonpioneer remodeling activity (Fig. 4B). We thus propose a mechanism of interdependent binding of ASCL1 and mSWI/SNF at active DREs, resulting in coregulation of chromatin remodeling and gene regulation (Fig. 6C,D).

## Discussion

We used genome-wide genetic and epigenetic analyses to investigate the pioneer function of ASCL1 in human neurogenesis. By analyzing our human neural differentiation culture system by scRNA-seq, we identified a transient population of ASCL1-dependent progenitors that rely on ASCL1 for cell cycle exit and neurodifferentiation. Although a progenitor population has already been termed “transitional progenitors” in an earlier study (Earley et al. 2021), the neuronal differentiation protocol was very different and the cell cluster, enriched in neurogenic transcription factors including but not defined by ASCL1, likely represents a very different cell stage. We propose that ASCL1-dependent transitional progenitors are a transient cell type defined by ASCL1 expression and its *cis*-regulatory effect on the transcriptional landscape.

ASCL1 regulates hundreds of genes in neural differentiation cultures, and we found evidence for direct binding of DREs in transitional progenitors. Investigating ASCL1's pioneer transcription factor function using a knockout model to characterize the unbound state, we identified two groups of regulated loci: those where ASCL1 acts as a classical pioneer TF, and another group where it binds permissive DNA and is required to promote further chromatin changes. We appreciate that chromatin accessibility is a continuum rather than a binary state; hence, unbiased statistical methods were used to define open and closed chromatin. Furthermore, we cannot rule out that partially open DNA in *ASCL1* KO bulk ATAC-seq data sets represents an averaging of accessible and inaccessible sites at the individual cell and locus level, which would need to be resolved by accessibility profiling at the single-cell level. However, these later sites are reminiscent of the “nonclassical pioneer factor” class proposed by Minderjahn et al. (2020) to define PU.1, which is unable to access nucleosomal target sites but, when overexpressed, can remodel chromatin and redistribute partner TFs in a mSWI/SNF complex-dependent manner. Recent studies have used inducible expression models to investigate pioneer function and have proposed that pioneer activity can be a quantitative trait of any transcription factor, dependent on TF abundance and genomic environment (Hansen and Cohen 2022). In contrast, here we have identified both pioneer and nonpioneer activities for endogenously expressed ASCL1 in conditions mimicking physiological development. We use the term “nonpioneer chromatin remodeling activity” to refer to the role of ASCL1 at sites where it affects chromatin state after binding permissive DNA. The diversity of ASCL1's roles at different locations emphasizes the context-dependent character of transcription factor activity.

Cooperative binding of chromatin remodeling complexes and pioneer transcription factors has come under particular attention in recent years. mBAF SWI/SNF has been implicated as an interactor with other pioneer factors, such as OCT4, GATA1, GATA3, and KLF4 (Hu et al. 2011; Takaku et al. 2016; King and Klose 2017; Moonen et al. 2022). In mouse ES cells, the SMARCA4 ATPase subunit of mSWI/SNF is required at OCT4-dependent sites to regulate chromatin accessibility at DREs and to allow binding of other pluripotency-associated transcription factors (King and Klose 2017). Pioneer factor KLF4 and SMARCA4-containing SWI/SNF complexes co-occupy active enhancer regions in endothelial cells, where KLF4 regulates chromatin accessibility of vasculo-protective genes in response to laminar sheer stress (Moonen et al. 2022).

We identified a cooperative function of bHLH transcription factor ASCL1 and mSWI/SNF chromatin remodeling complexes at DREs in regulating chromatin structure and in promoting neurogenesis at a key transitional stage (Fig. 5). We found evidence for codependent DNA binding, with interference with one binding partner affecting binding of the other at coregulated sites, indicating that they are not redundant in nucleosome displacement activity (Fig. 6). Cooperative interaction between ASCL1 and ATPase-active SWI/SNF is greatest at regulatory elements where ASCL1 acts as a nonpioneer chromatin remodeler (53.1% of sites). Conversely, at loci where ASCL1 acts as a classical pioneer factor, although a subset of the loci is cobound by mSWI/SNF (12.7%), they do not require its presence to elicit changes in chromatin structure. We observed that interfering with chromatin remodeling (*ASCL1* KO or mSWI/SNF activity inhibition) results in significant reduction in binding of the cooperative partner, though residual binding of mSWI/SNF in the absence of ASCL1 is presumably sufficient for some degree of accessibility. This suggests that the ASCL1–mSWI/SNF interaction may act to stabilize mSWI/SNF at its targets and that, in the absence of ASCL1, mSWI/SNF releases from DNA more readily.

Using molecularly defined active regulatory regions (i.e., with ATAC-seq and H3K27ac ChIP-seq signals), we computationally predicted enhancer–gene pairs regulated by the ASCL1–mSWI/SNF interaction. Genes coregulated by this interaction and where ASCL1 acts as a nonpioneer chromatin remodeler were enriched for pathways and ontology terms associated with cell differentiation, brain development, and neurotransmitter processes, supporting a role in directing neural differentiation (Supplemental File S5). There are fewer coregulated genes where ASCL1 acts as a classical pioneer TF, yet these include important neuronal genes, including *BCL11B* (Fig. 6C), encoding transcription factor BCL11B/CTIP2 expressed in deep-layer cortical neurons (Simon et al. 2020). It is likely that, similar to OCT4–SMARCA4 interaction regulating pluripotency (King and Klose 2017), the pioneer ASCL1–SWI/SNF interaction may facilitate binding of other TFs important for neural differentiation, though further studies would be required to confirm that.

One possibility for the distinct requirements of SWI/SNF as an interacting partner at classical pioneer and nonpioneer sites of chromatin remodeling is that different loci may have architectural constraints that either require or limit ASCL1–DNA interaction. ASCL1, in a heterodimer with bHLH E proteins, binds an E-box motif with two degenerate central nucleotides (5′-CANNTG-3′) (Soufi et al. 2015; Fernandez Garcia et al. 2019). We did not find evidence for differences in binding motifs at sites of classical and nonpioneer chromatin remodeler activity; therefore, we speculate that, as per the previously reported ASCL1 pioneer function (Wapinski et al. 2013), cooperative binding between ASCL1 and mSWI/SNF may be determined by factors such as histone modifications, which we do not explore here. A limitation of the study is that molecular characterization of the system is not performed at the single-cell level apart from scRNA-seq. Since ASCL1 is expressed primarily in transitional progenitors, we can assume the ChIP-seq binding profiles are specific to these cells. However, the ATAC-seq profile reflects the whole cultured cell population and, moreover, as with other DNA accessibility techniques, ATAC-seq does not perform well on detection of partially unwrapped nucleosomes (Sung et al. 2014). Thus, it is possible that the nonpioneer chromatin remodeler ASCL1 repertoire and cooperative binding of mSWI/SNF at those sites represent heterogeneous states that will be more readily deciphered with higher-resolution analysis (Buenrostro et al. 2015; Hainer et al. 2019; Harada et al. 2021). As we found cooperative binding for only a small subset of ASCL1 classical pioneer targets, we note that we did not investigate other chromatin remodelers than mSWI/SNF, which may act with ASCL1 in regulating chromatin structure at sites of classical pioneer activity.

Similar to previous studies in reprogramming of fibroblasts to neurons (Wapinski et al. 2017), we identified a repressive role for ASCL1, which is primarily at sites where it acts as a nonpioneer chromatin remodeler (Supplemental Fig. S4A,B). Motif analysis at these sites is more enriched for homeobox and HMG binding motifs than bHLH motifs (Supplemental File S4E), suggesting cooperative binding with different transcriptional regulators. Further investigations will be required to determine mechanisms and interactors mediating the repressive role of ASCL1.

The use of a constitutive knockout of ASCL1 rather than conditional ablation may also be a limitation of the study. However, the observation that the chromatin accessibility landscape of knockout cells at ASCL1 peak expression (DIV24) is similar to that of wild-type cells at a time point preceding significant ASCL1 expression (DIV20; prebound state) suggests that constitutive loss does not have a significant effect prior to NOTCH inhibition and the sharp increase in ASCL1 expression at DIV23–24. Conversely, while we ablated mSWI/SNF enzymatic activity preserving protein expression (Papillon et al. 2018; Iurlaro et al. 2021), we cannot exclude ATPase-independent roles for mSWI/SNF, which might be required at sites of ASCL1 classical pioneer activity bound by mSWI/SNF (Jordán-Pla et al. 2018). Having focused our attention on the cooperative functions of ASCL1 and mSWI/SNF, further investigations are also required to explore whether the roles of mSWI/SNF in the transition from neural progenitors to neurons in mouse neurogenesis are replicated in humans (Yoo et al. 2009; Staahl et al. 2013) and whether these roles reflect predominantly mSWI/SNF's interaction with ASCL1 or also interactions with other neurogenic factors.

The importance of ASCL1 and BAF complex proteins to human development is highlighted by the genetic constraint for loss-of-function mutation in the human population. ASCL1 is moderately constrained for loss of function (observed/expected [o/e] metric of 0.13 [one out of eight]) in the gnomAD v2.1.1 database), with no observed homozygotes (Karczewski et al. 2020). Missense and in-frame ASCL1 variants were previously suggested in central hypoventilation syndromes; however, convincing pathogenicity evidence is lacking. Complete loss of mSWI/SNF subunits is also not observed in the same population database. SMARCC1 and SMARCC2 are required for forebrain development in mouse models, with evidence for essential roles in proliferation, differentiation, cell cycle progression, cell survival, and layer formation (Narayanan et al. 2015). They are confirmed (SMARCC2) (Machol et al. 2019) or candidate (SMARCC1) (Furey et al. 2018) genes for neurodevelopmental phenotypes with heterozygous mutations. Various other BAF subunits have been implicated in neurodevelopmental disorders (Kosho et al. 2014; Pulice and Kadoch 2016; Sokpor et al. 2017).

Our findings of a regulatory interaction between ASCL1 and mSWI/SNF chromatin remodelers in controlling the neurogenic process may have implications beyond ASCL1's role in neural development. Glioblastomas respond to overexpression of ASCL1 by terminally differentiating proliferating cells, thus restricting tumor expansion (Park et al. 2017). The identification of mBAF SWI/SNF dependency at neurogenic regulatory loci may indicate a targetable vulnerability in these cancers.

## Materials and methods

### iPSC culture and differentiation

Experiments were performed using the Human Induced Pluripotent Stem Cell Initiative (HipSci; https://www.hipsci.org) Kolf2C1 line (clone C1 of parental line HPSI0114ikolf2), a kind gift from William T. Skarnes at the Wellcome Sanger Institute. The stem cells were maintained at 37°C and 5% CO_2_ under feeder-free conditions on Geltrex-coated (Thermo Fisher Scientific) plasticware (Corning) in E8 media (Thermo Fisher Scientific) plus 100 U/mL penicillin/streptomycin (Thermo Fisher Scientific). Given that SMAD inhibition has previously been shown to recapitulate in vivo neurogenesis in the temporal sequence of production of different cortical projection neurons and the functional integration and transcriptional identity of different cell types (Shi et al. 2012; Handel et al. 2016), the iPSCs were differentiated into cortical neurons in two-dimensional adherent cultures using a dual-SMAD inhibition protocol (modified from Chambers et al. 2009; Shi et al. 2012). Briefly, the iPSCs were washed with Dulbecco's phosphate-buffered saline (DPBS; Thermo Fisher Scientific), dissociated in 0.5 mM ethylenediaminetetraacetic acid (EDTA; pH 8.0; Invitrogen) in DPBS, and plated on Geltrex-coated plates in E8 in a 2:1 ratio on day −1. After 24 h (day 0), the culture reached 80%–100% confluency, at which point the medium was replaced with neural induction medium (N2B27; 1:1 mixture of N2 medium and B27 medium) (composition listed in Supplemental Table S2) supplemented with 10 mM SB31542 (Abcam) and 10 nM LDN193189 (Stem Cell Technologies). The neural induction medium was replaced daily for 7 d. On day 7, the neuroepithelial cells were washed with Hanks’ balanced salt solution (HBSS; Thermo Fisher Scientific), detached using Accutase (Sigma-Aldrich), and replated on Geltrex-coated plates in N2B27 with 10 mM Y-27632 ROCK inhibitor (Tocris). The ROCK inhibitor was removed after 24 h (day 8), and the neural induction medium was replaced daily until day 12. On day 12, the neural progenitors were dissociated in Accutase and replated on Geltrex-coated plates in N2B27 with 10 mM Y-27632 ROCK inhibitor. ROCK inhibitor was removed on day 13, and cells were maintained in daily-changed N2B27 until day 23 (with 1:2 passaging on days 15–16 and 19–20 as already described for days 7 and 12). On day 23, the medium was changed to B27 only supplemented with 10 mM DAPT (Cambridge Bioscience). On days 25 and 27, half of the media was replaced with fresh B27 supplemented with 10 mM DAPT. From day 30 onward, the neurons were maintained in B27 medium only, replacing only half of the B27 media volume every week.

Because the Kolf2C1 line was subsequently identified as harboring a somatic ARID2 frameshift mutation (Skarnes et al. 2019), we performed the neural differentiation protocol above on Kolf2C1, the gene-corrected derivative KOLF2.1J (a kind gift from William T. Skarnes, The Jackson Laboratory for Genomic Medicine), and independent HipSci line Kucg2 (HPSI0214i-kucg_2) and analyzed genome wide transcriptomes by RNA-seq at DIV24 where most of our experimental work is conducted. We found that the corrected KOLF2.1J and Kolf2C1 have similar transcriptomes, clustering tightly together and apart from the Kucg2 line on principal component analysis (Supplemental Fig. S5). This finding indicates that transcriptional differences attributable to the single gene change were minimal in comparison with those induced by genetic background (Kilpinen et al. 2017), and we thus proceeded with the Kolf2C1 line for which our neuralization protocol had been optimized.

### Generation of ASCL1 knockouts

ASCL1 knockout Kolf2C1 cells were generated with CRISPR/Cas9 methods modified from Bruntraeger et al. (2019) and using two previously validated *ASCL1* crRNA sequences (Liu et al. 2016). Briefly, wild-type Kolf2C1 cells were supplemented with 10 mM ROCK inhibitor 1 h prior to nucleofection. For each *ASCL1* crRNA, 200 mM crRNA and 200 mM tracrRNA (IDT) were mixed in IDT duplex buffer (IDT) in a 1:1:2.5 ratio and then hybridized by heating for 2 min to 95°C before returning to room temperature to form sgRNAs. The two sgRNAs were then combined 1:1, and 5 mL of this was mixed with 20 μg of Alt-R S.p. HiFi Cas9 nuclease V3 (IDT) and incubated at room temperature for 30 min to form Cas9 ribonucleoprotein (RNP) complexes. Concurrently, cells were collected with Accutase supplemented with 10 mM ROCK inhibitor, washed twice in DMEM F-12 (Thermo Fisher Scientific), and then strained through a 40-mm cell strainer. Cells (10^6^) were then resuspended in 100 mL of P3 buffer (Lonza) and mixed with the prepared Cas9-RNPs and 5 mL of EP enhancer (IDT). This was then nucleofected on an Amaxa 4D nucleofector using program CA137 (Lonza) and plated into a Synthemax-coated (Sigma-Aldrich) six-well plate in E8 plus ROCK inhibitor. After recovery, cells were plated at single-cell density to obtain colonies of single clones using CloneR-supplemented (Stem Cell Technologies) media, following the manufacturer's instructions, and manually picked into 96-well plates. Single clones were screened for desired edits by targeted amplicon high-throughput sequencing (MiSeq) using previously published PCR primers (Liu et al. 2016) with appended Illumina sequencing adaptors and barcodes. Clones with biallelic deletions inducing a frameshift (134-bp deletion, ENST00000266744.4:c.92_225del, ENSP00000266744.3:p.Phe31TrpfsTer81) were expanded, and knockout was confirmed by Western blotting.

### RNA extraction, cDNA synthesis, and qRT-PCR

Samples were collected in RLT lysis buffer (Qiagen) added directly to the DBPS-rinsed cell culture plates (Thermo Fisher Scientific). RNA was then extracted using the RNeasy micro kit (Qiagen) according to the manufacturer's protocol, with 15-min on-column digestion with RNase-free DNase I (Qiagen) to eliminate genomic DNA. RNA was converted to cDNA using the Maxima first strand cDNA synthesis kit with dsDNase (Thermo Fisher Scientific) using reverse transcriptase with a mix of random hexamer and oligo(dT) 18 primers, according to the manufacturer's protocol. qRT-PCR reactions were prepared with TaqMan Universal qRT-PCR master mix (Thermo Fisher Scientific) and commercially designed primer probes (Supplemental Table S3) following the manufacturer's protocol. Reactions were prepared in triplicate and run on the LightCycler 480 II thermal cycler (Roche). Data were exported from proprietary Roche software and subjected to statistical testing in Excel (Microsoft) based on the −2^−ΔΔ*Ct*^ or Livak method (Livak and Schmittgen 2001). Briefly, gene products from the three technical replicates were averaged, followed by normalization against *HPRT* or *UBC* to generate the Δ*Ct* values, which were then compared with the day 0 values to generate the ΔΔ*Ct* values. qRT-PCR analysis was performed on samples obtained from a minimum of three independent experiments. Data were graphed using GraphPad Prism software.

### Western blotting

Cells were washed with DPBS and lysed in Pierce IP lysis buffer (Thermo Fisher Scientific) supplemented with 1× protease inhibitor cocktail (Thermo Fisher Scientific), 1× EDTA (Thermo Fisher Scientific), and 1× phosphatase inhibitor cocktail (Thermo Fisher Scientific). Cells were scraped off the plates and lysed for 20 min at 4°C under rotation, followed by centrifugation at 17,000*g* for 20 min. Supernatant was collected in a new tube and stored on ice for quantification. A bicinchoninic acid assay (Pierce BCA protein assay kit, Thermo Fisher Scientific) was used to quantify the protein extract supernatant according to the manufacturer's protocol. Bovine serum albumin (BSA; Thermo Fisher Scientific) was used to generate a standard curve, and color change was then quantified using the EnSight multimode plate reader (Perkin Elmer) and analyzed using the proprietary software. Following quantification, protein was stored at −80°C until analyzed by Western blot or subjected to immunoprecipitation. Samples were then prepared for sodium dodecyl sulfate polyacrylamide gel electrophoresis (SDS-PAGE) by dilution with 2× (Sigma) or 5× (in-house-made) Laemmli sample buffer and incubation for 5 min at 95°C. Denatured samples were run on 4%–15% polyacrylamide gels (Bio-Rad) in 1× Tris-glycine-SDS (TGS) running buffer (Bio-Rad) at 120–130 V. A polyvinylidene fluoride (PVDF) membrane (Bio-Rad) was used for sample transfer using the Trans-Blot Turbo transfer system (Bio-Rad) before blocking in 5% milk (Marvel) in Tris-buffered saline Tween (TBS-T; Bio-Rad) for 60 min. Membranes were incubated with primary antibodies (Supplemental Table S4) diluted in 5% milk (Marvel) in TBS-T overnight at 4°C with rocking. The next day, membranes were washed in TBS-T, followed by incubation in secondary antibodies (Supplemental Table S4) diluted in 5% milk in TBS-T for 60 min at room temperature. TBS-T washes were performed again, and signal was generated using enhanced chemiluminescence (ECL) substrate (Amersham) as per the manufacturer's instructions. A Hyperfilm ECL (Amersham) was developed for signal detection in a dark room.

### Coimmunoprecipitation

For coimmunoprecipitation (co-IP) experiments, primary antibodies (Supplemental Table S4) were added to the protein supernatants and incubated with rotation for 2 h at 4°C. At the same time, Sepharose coupled with protein G (Sigma) was blocked with 5% BSA (Sigma) in precooled DPBS (Thermo Fisher Scientific) for 2 h with rotation at 4°C. After three washes with cold DPBS, Sepharose coupled with protein G was added to the protein lysate–antibody mixture and incubated for 90 min at 4°C under rotation. The protein–antibody–Sepharose mixture was then washed five times in Pierce IP lysis buffer (Thermo Fisher Scientific) and resuspended in 2× Laemmli sample buffer (Sigma). Samples were then incubated for 5 min at 95°C and stored at −80°C until Western blot analysis.

### Immunofluorescence

Cells were plated on Geltrex-coated glass coverslips. Cultured cells were fixed in 4% paraformaldehyde (PFA) in PBS (Alfa Aesar) for 10 min at room temperature followed by two washes in DPBS.

Human fetal tissue from terminated pregnancies was obtained from the joint Medical Research Council/Wellcome Trust Human Developmental Biology Resource (HDBR; https://www.hdbr.org) (Gerrelli et al. 2015), which has been granted approval to function as a Research Tissue Bank (by the National Research Ethics Service [NRES]) under research ethics committee approvals 18/LO/0822 and Newcastle 18/NE/0290. For immunostaining experiments, the fetal brains were fixed with 4% PFA in PBS (Alfa Aesar) for at least 24 h at 4°C. After fixation, brains were dehydrated in graded ethanol washes and embedded in paraffin, before being cut and mounted onto slides (the Experimental Histopathology Laboratory, the Francis Crick Institute).

Both cells and tissue were subjected to antigen retrieval in 10 mM Na citrate: boiled 10 min at 95°C for adherent cells, and 30 sec in the microwave for brain tissue. Samples were permeabilized in 0.1% Triton-PBS for 10 min at room temperature with rocking, blocked with 10% normal donkey serum (NDS; Jackson ImmunoResearch) in 0.1% Triton-PBS for 1 h at room temperature with rocking, and subsequently incubated in primary antibodies (Supplemental Table S4) diluted in 10% NDS in 0.1% Triton-PBS overnight at 4°C with rocking. The next day, samples were washed three times in PBS and incubated in secondary antibodies (Supplemental Table S4) and 10 g/mL DAPI (Sigma) diluted in 10% NDS in 0.1% Triton-PBS for 90 min at room temperature with rocking. Following three washes in PBS, samples were mounted in VectaShield mounting medium (Vector Laboratories). Immunofluorescence was performed on a minimum of three biological replicates from independent in vitro neuronal differentiations.

### Proximity ligation assay

Proximity ligation assay (PLA) was performed using the Duolink in situ Red Started kit mouse/rabbit (Sigma Aldrich) according to the manufacturer's instructions. Briefly, cells or human fetal brain tissue were subjected to fixation, antigen retrieval, and permeabilization as described above. Samples were then blocked in Duolink blocking solution for 60 min at 37°C, followed by incubation in primary antibodies (Supplemental Table S4) diluted in Duolink antibody diluent overnight at 4°C. The next day, samples were washed twice in Duolink wash buffer A, followed by incubation with PLA Plus and Minus probes for 1 h at 37°C, ligation using the Duolink ligase diluted in the 5× Duolink ligation buffer for 30 min at 37°C, and amplification using Duolink polymerase diluted in the 5× Duolink amplification buffer for 100 min at 37°C. All samples were washed twice in Duolink wash buffer A and once in Duolink wash buffer B, followed by mounting in Duolink in situ mounting medium with DAPI.

### Image acquisition

Imaging was performed using a laser scanning TCS SP5 II confocal microscope (Leica Microsystems) at a *z*-section thickness of 1 μm. The same settings were applied to all images. Images were visualized with Fiji (Schindelin et al. 2012). Analysis of images for PLA was carried out in Fiji (Schindelin et al. 2012): Maximum-intensity *Z* projection was performed followed by nucleus segmentation using Stardist (Schmidt et al. 2018). Intranuclear dots were detected via Fiji's Find Maxima function. Dots occurring within segmented nuclei were then counted automatically on a per-nucleus basis.

### Intracellular flow cytometry

Twenty-four hours after exposure to the NOTCH inhibitor DAPT (Tocris), cells were washed in DPBS and dissociated with Accutase (Sigma) using a P1000 pipette. Once detached, cells were collected into 15-mL tubes with 4 vol of DMEM-F12 (Thermo Fisher Scientific) and pelleted at 300*g* for 3 min. Cells were resuspended in DPBS, pelleted, resuspended in Live/Dead fixable near-IR dead cell stain (Invitrogen) as per the manufacturer's instructions, and left to incubate for 30 min at room temperature protected from light. Cells were subsequently pelleted and washed once in DPBS, before fixation in 4% paraformaldehyde in PBS. Following 10-min incubation, cells were washed with DPBS by dilution, pelleted, and resuspended in PBS before storage at 4°C for future analysis. On the day of analysis, cells were transferred for staining into V-bottomed 96-well plates (Corning). Samples were pelleted at 1000*g* for 4 min at 4°C, resuspended in 100 μL of primary antibodies (Supplemental Table S4) diluted in PBS plus 0.2% Triton X-100 plus 3% donkey serum (Jackson), and incubated overnight at 4°C. The next day, samples were washed by dilution with 100 μL of PBS, pelleted, and resuspended in 100 μL of PBS to complete the wash. After washing, cells were pelleted and then resuspended and incubated in 100 μL of secondary antibodies (Supplemental Table S4) plus 1 μg/mL DAPI for 1 h. One additional wash was performed before transferring into cell strainer-capped tubes (Falcon) for acquisition on a Fortessa flow cytometer (BD) using FACSDiva software. Analysis was subsequently performed in FlowJo.

### CRISPR/Cas-9 targeting of neuronal cultures

DIV21 neural progenitor cultures were electroporated with both SMARCC1 and SMARCC2 targeting plasmids (Supplemental Table S4) using program A-033 on the Lonza Nucleofector 2b device with the mouse neural stem cell nucleofector kit. Successfully targeted neural progenitors were then selected using 400 g/mL neomycin and 150 g/mL hygromycin for 48 h.

### RNA-seq and analysis

Libraries were prepared using the KAPA mRNA polyA HyperPrep kit (Illumina) and subsequently sequenced on the Illumina HiSeq4000 platform (Advanced Sequencing Facility, the Francis Crick Institute) to generate 100-bp paired-end reads.

For sequence analysis, adapter trimming was performed with CutAdapt (Martin 2011) with parameters “–minimum-length = 25 –quality-cutoff = 20 -a AGATCGGAAGAGC.” The RSEM package (Li and Dewey 2011) in conjunction with the STAR alignment algorithm (Dobin et al. 2013) was used for the mapping and subsequent gene-level counting of the sequenced reads with respect to the human UCSC hg19 genome and annotation (UCSC) (Karolchik et al. 2004) downloaded from AWS iGenomes (https://github.com/ewels/AWS-iGenomes). The parameters passed to the “rsem-calculate-expression” command were “–star –star-gzipped-read-file –star-output-genome-bam –forward-prob 0.” Differential expression analysis was performed with the DESeq2 package (Love et al. 2014) within the R programming environment (v3.3.1) . An adjusted *P*-value of ≤0.05 and a fold change of ≥1.5 were used as the significance threshold for the identification of differentially expressed genes.

For functional annotation, the online tool DAVID version 2021 bioinformatics resource (https://david.ncifcrf.gov/summary.jsp) was used (Sherman et al. 2022). A background gene list was generated from genes expressed in wild-type DIV24 cultures where the RSEM-computed expected count was ≥10 for bulk RNA-seq in at least one replicate. The “GOTERM_BP_DIRECT” and “GOTERM_MF_DIRECT” functional annotation terms were selected to identify statistically enriched gene ontology annotations, and “REACTOME_PATHWAY” was selected for overrepresentation of biological pathways within sets of gene IDs associated with differentially expressed transcripts and to calculate associated Benjamini–Hochberg-adjusted *P-*values.

### ChIP-seq

The ChIP-seq protocol was modified from Sullivan and Santos (2020). Cells were fixed with 2 nM di(N-succimidyl) glutarate (Sigma-Aldrich) in DPBS (Thermo Fisher Scientific) for 45 min at room temperature on a rocking platform. Three DPBS washes were then performed, before a second 10-min fixation in 1% methanol-free formaldehyde solution (Thermo Fisher Scientific) in DPBS at room temperature with rocking. The formaldehyde fixation was stopped by adding 1 mL of 1.25 M glycine (Sigma-Aldrich), followed by a 5-min incubation on the rocking platform at room temperature. Cells were scraped off and pelleted by centrifugation at 800*g* for 5 min at 4°C. After three washes with ice-cold DPBS, the cell pellet was snap-frozen in liquid nitrogen and stored at −80°C until processing. To isolate nuclei, pellets were resuspended in 300 mL of SDS lysis buffer (Supplemental Table S2) containing 1× protease inhibitor cocktail (Thermo Fisher Scientific) and incubated for 30 min on ice. The cell suspension was transferred to a 1.5-mL Diagenode TGX tube (Diagenode) and sonicated for 75 cycles of 30 sec on and 30 sec off on high in a precooled Diagenode Bioruptor Plus sonication system. One milliliter of chromatin dilution buffer (Supplemental Table S2) containing 1× protease inhibitor cocktail was added to the cross-linked sheared chromatin and centrifuged at 14,000*g* for 30 min at 4°C. Sixty milliliters of soluble chromatin was stored at −20°C as input chromatin, while the remaining supernatant was transferred to a protein LoBind tube (Fisher Scientific) containing protein A or G Dynabeads (Thermo Fisher Scientific)–primary antibody (Supplemental Table S4) mix (previously incubated with rotation for 3 h at room temperature). The chromatin–antibody–Dynabeads solution was incubated overnight at 4°C with rotation. Using a magnetic holder to separate the Dynabeads, the supernatant was removed and sequentially washed for 5 min at 4°C under rotation with 1 mL of wash buffer A, wash buffer B, and wash buffer C and twice with TE buffer (Supplemental Table S2). One-hundred milliliters of elution buffer (Supplemental Table S3) was added after the final wash, followed by a 5-min incubation at 65°C. Dynabeads were separated using a magnetic holder, and the eluted DNA was transferred to a clean 1.5-mL tube. The elution step was repeated, resulting in 200 mL of final DNA. The input chromatin from day 1 was removed from the freezer, and the NaCl (Sigma-Aldrich) concentration was increased to 160 mM for all samples. RNase A (Thermo Fisher Scientific) was added to a final concentration of 20 μg/mL, and all samples were incubated overnight at 65°C to reverse cross-links and digest contaminating RNA. On day 3, EDTA concentration was increased for all samples to 5 mM (Sigma-Aldrich) followed by a 2-h incubation with 200 μg/mL proteinase K (Sigma-Aldrich) to digest proteins.

ChIP and input samples were purified using the Zymo Clean and Concentrator-5 kit (Zymo) according to the manufacturer's instructions. DNA fragment size and distribution were determined by Agilent TapeStation (Agilent) before DNA library preparation using the NEB Ultra II DNA library preparation kit for Illumina (New England Biolabs) as per the manufacturer's instructions. ChIP-seq samples were sequenced on the Illumina HiSeq4000 platform (Advanced Sequencing Facility, the Francis Crick Institute), and 100-bp single-end reads were generated.

### ChIP-seq analysis

The nf-core/ChIP-seq pipeline (Ewels et al. 2020; https://doi.org/10.5281/zenodo.3529400), written in the Nextflow domain-specific language (Di Tommaso et al. 2017), was used to perform the primary analysis of the samples in conjunction with Singularity (Kurtzer et al. 2017). The command used was “nextflow run nf-core/chipseq –input design.csv –genome hg19 min_reps_consensus 2 -profile crick -r 1.1.0”; where applicable, the “–single_end “ and “–narrow_peak” (for ASCL1 immunoprecipitations) or “–broad_peak” (for SMARCB1, H3K4me3, and H3K27ac immunoprecipitations) parameters were used. To summarize, the pipeline performs adapter trimming (Trim Galore! [https://www.bioinformatics.babraham.ac.uk/projects/trim_galore]), read alignment (BWA [Li and Durbin 2009]), filtering (SAMtools [Li et al. 2009], BEDTools [Quinlan and Hall 2010], BamTools [Barnett et al. 2011], pysam [https://github.com/pysam-developers/pysam], and picard-tools [http://broadinstitute.github.io/picard]), normalized coverage track generation (BEDTools [Quinlan and Hall 2010] and bedGraphToBigWig [Kent et al. 2010]), peak calling (MACS [Zhang et al. 2008]), annotation relative to gene features (HOMER [Heinz et al. 2010]), consensus peak set creation (BEDTools [Quinlan and Hall 2010]), differential binding analysis (featureCounts [Liao et al. 2014], R [[R Core Team 2017], and DESeq2 [Love et al. 2014]), and extensive QC and version reporting (MultiQC [Ewels et al. 2016], FastQC [https://www.bioinformatics.babraham.ac.uk/projects/fastqc], preseq [Daley and Smith 2013], deepTools [Ramírez et al. 2016], and phantompeakqualtools [Landt et al. 2012]). All data were processed relative to the human UCSC hg19 genome build (UCSC) (Karolchik et al. 2004) downloaded from AWS iGenomes (https://github.com/ewels/AWS-iGenomes). Peak annotation was performed relative to the same GTF gene annotation file used for the RNA-seq analysis. Tracks illustrating representative peaks were visualized using the IGV genome browser (Robinson et al. 2011).

Motif enrichment analyses of ChIP-seq peak data sets were performed using HOMER (Heinz et al. 2010) findMotifsGenome with default parameters and region size set to 200 bp (±100 bp adjacent to peak center): “findMotifsGenome.pl <peak/BED file> <genome> <output directory>- size 200.”

### ATAC-seq

ATAC-seq sample preparation was performed using previously established protocols (Buenrostro et al. 2013, 2015; Corces et al. 2017). Briefly, 50,000 cells at each stage were isolated and pelleted at 500*g* for 5 min at 4°C; lysed in 50 mL of ice-cold ATAC resuspension buffer (RSB) (Supplemental Table S2) containing 0.1% NP40 (Sigma-Aldrich), 0.1% Tween-20 (Sigma-Aldrich), and 0.01% digitonin (Promega); and incubated for 3 min on ice. The lysis reaction was stopped with 1 mL of ice-cold ATAC-RSB containing 0.1% Tween-20 (Sigma-Aldrich), and the nucleus extracts were isolated by centrifugation at 500*g* for 10 min at 4°C. The cell pellet was then resuspended in 50 mL of transposition reaction mix (25 mL of 2× TD buffer [Illumina], 2.5 mL transposase [Illumina], 16.5 mL of DPBS [Thermo Fisher Scientific], 0.5 mL of 1% digitonin [Promega], 0.5 mL of 10% Tween-20 [Sigma-Aldrich], 5 mL of water [Thermo Fisher Scientific]) and subsequently incubated for 30 min at 37°C in a thermomixer with 1000 rpm mixing. Transposed DNA was purified using the Zymo Clean and Concentrator-5 kit (Zymo) according to the manufacturer's protocol and eluted in 21 mL of elution buffer. Five milliliters of the cleaned transposed DNA was used for library amplification for 12 cycles using the NEBNext HiFi 2× PCR master mix (New England Biolabs) and previously designed ATAC-seq barcoded primers (Supplemental Table S5; Buenrostro et al. 2013). PCR reactions were cleaned up with KAPA pure beads (Roche) at 1.8× beads versus sample ratio. Prior to sequencing, DNA fragment size and distribution and library concentration were determined by Agilent TapeStation (Agilent) and QubitTM dsDNA HS assay (Life Technologies), respectively. ATAC-seq samples were subsequently sequenced on the Illumina HiSeq4000 platform (Advanced Sequencing Facility, the Francis Crick Institute), and 100-bp paired-end reads were generated.

### ATAC-seq analysis

The nf-core/atacseq pipeline (https://doi.org/10.5281/zenodo.3529420) (Ewels et al. 2020), written in the Nextflow domain-specific language (Di Tommaso et al. 2017), was used to perform the primary analysis of the samples in conjunction with Singularity (Kurtzer et al. 2017). The command used was “nextflow run nf-core/atacseq –design design.csv –genome hg19 -min_reps_consensus 1 -profile crick -r 1.1.0.” The nf-core/atacseq pipeline uses processing steps similar to those described for the nf-core/chipseq pipeline in the previous section but with additional steps specific to ATAC-seq analysis such as the removal of mitochondrial reads.

For peak intersection, BEDtools intersectBed was used to identify genomic intervals overlapping by 1 bp in BED files listing coordinates of consensus peak sets: “bedtools intersect -a file1.bed -b file2.bed > output.txt.”

### Single-cell RNA-seq and analysis

Wild-type and *ASCL1* KO cells were differentiated concurrently as described above. On the day of harvest, adherent cell cultures were washed twice with DPBS, incubated at 37°C in Accutase (Sigma-Aldrich), and manually dissociated by pipetting with a P1000. Two volumes of DMEM F-12 was added, and cells were passed through a 40-μm cell strainer to achieve a single-celled suspension before counting on a NucleoCounter NC-200. Cells (1.5 × 10^6^) were then centrifuged at 255*g* for 2 min. The pellet was resuspended in 750 mL of 0.22-μm-filtered 1% BSA in DPBS for a theoretical concentration of 2000 cells/mL. This suspension was then counted. Ten-thousand cells were loaded onto the Chromium X (10X Genomics) to generate single-celled gel beads in emulsion (GEMs) for library preparation. Dual-index Chromium single-cell 3′ v3.1 chemistry was used according to the manufacturer's instructions, using 11 cycles for the cDNA amplification and 14 cycles for the library PCR. Libraries were then sequenced on a NovaSeq 6000 (Illumina).

#### Preprocessing

Feature quantification of the spliced and unspliced reads was performed using Alevin (Srivastava et al. 2019), as recommended by Soneson et al. (2021). Reads mapped to the human reference genome (ENSEMBL GRCh37, release 75). The quantification was carried out in two stages. In the first pass, Cell Ranger single-cell software suite from 10X Genomics was used to identify a filtered whitelist of cell barcodes. This whitelist of cell barcodes was used as input for Alevin quantification in second pass. This helped use the Cell Ranger's default filtering criteria for selection of cell barcodes for downstream analysis. Further analysis was carried out using Seurat package (Butler et al. 2018; Stuart et al. 2019; Hao et al. 2021) in R-4.0.0 ([R Core Team 2020). A sample-specific threshold for low-quality cells was identified using median absolute deviation (MAD) measures for cells expressing >3 MAD of mitochondrial gene expression along with <3 MAD of total number of detected features/genes. Suspected doublet cells were identified using scDblFinder v1.4.0 (Germain et al. 2021) and were excluded from subsequent analysis. Using default parameters within the Seurat package (Butler et al. 2018; Stuart et al. 2019; Hao et al. 2021), each sample was normalized, and the variance was stabilized across cells using the SCTransform v0.3.3 (Hafemeister and Satija 2019) with glmGamPoi (Ahlmann-Eltze and Huber 2021) method.

#### Integration across samples

After filtering of cells/clusters based on mitochondrial gene expression and the number of detected features, we performed the following sets of integration of the samples using the standard workflow from the Seurat package (Butler et al. 2018; Stuart et al. 2019; Hao et al. 2021). These included integration of samples from individual sample groups (WT cells with and without DAPT treatment and *ASCL1* KO cells with DAPT treatment) and by DAPT treatment or genotype: (1) WT cells with and without DAPT treatment (Supplemental Fig. S1B) and (2) WT cells and *ASCL1* KO cells with DAPT treatment (Supplemental Fig. S2C). After data normalization and variable feature detection in the individual samples using SCTransform (see above; Hafemeister and Satija 2019), anchors were identified using the “FindIntegrationAnchors()” function, and data sets were integrated with the “IntegrateData()” across 30 dimensions for 3000 integration features identified from the data sets. We then performed PCA on the integrated data, and the first 20 principal components were used to create a shared nearest neighbor (SNN) graph using the “FindNeighbors()” function. This was used to find clusters of cells showing similar expression using the “FindClusters()” function across a range of clustering resolution parameters (from Seurat package; 0.2–1.4 in 0.2 increments). For data visualization, dimensional reduction of the integrated data was achieved using UMAP with 20 principal components and with cosine correlation metric. For further downstream analysis, the raw RNA counts of the integrated data were used after normalization with the “LogNormalize” method.

For the integrated data set of WT cells treated with DAPT, clustering resolution parameter of 1.0 (Fig. 1B) was selected based on visual inspection of known marker genes (Fig. 1F; gene list is in Supplemental Table S6) that are enriched in individual clusters. Cell identity was determined based on the module score identified for these marker genes using the “AddModuleScore()” function. Using these integrated data as a reference, cell identities for the *ASCL1* KO data set were identified with the help of transfer anchors obtained using the first 20 principal components of SCTransform-normalized (Hafemeister and Satija 2019) features (Fig. 3A).

For the integrated data sets of (1) all WT cells with or without DAPT (Supplemental Fig. S1B) and (2) WT and *ASCL1* KO cells with DAPT treatment (Supplemental Fig. S2C), cell identities were determined by visual inspection based on their module scores of the same marker genes, like above.

#### RNA velocity estimation

The spliced and unspliced read counts from the integrated Seurat object was used as input into scVelo (Bergen et al. 2020) to calculate RNA velocity values for each gene of each cell. scVelo was used in the “dynamical” mode with default settings. The resulting RNA velocity vector was embedded into the PCA and UMAP space by translating the RNA velocities into likely cell transitions using cosine correlation to compute the probabilities of one cell transitioning into another cell. We identified driver genes (i.e., those genes that show dynamic behavior) as those genes with a fit likelihood in the dynamical model >0.3. We also used PAGA (Wolf et al. 2019) to perform trajectory inference, for which directionality was inferred from the RNA velocities.

#### Calculation of similarities between scRNA-seq data sets

To compare the scRNA-seq data set from DIV24 WT DAPT-treated cells with a publicly available human fetal brain data set, we used first-trimester forebrain samples (PCW 5–10) from Braun et al. (2022), obtained in .h5 format from https://github.com/linnarsson-lab/developing-human-brain (Supplemental Table S7). The data were subsetted and converted into an AnnData (v0.8.0) (Virshup et al. 2021) object in Python (version 3.9.16). Using SeuratDisk (v0.0.09020), the h5ad object was converted to a Seurat object. Using the published data set as a reference, the Bioconductor package clustifyr (v1.10.0) (Fu et al. 2020) was used to assign cluster labels from the reference to the DIV24 WT DAPT query data set. Clustifyr builds a scRNA-seq reference by averaging per-cell expression data for each cluster (“clusters” meta data column in Braun et al. 2022) from the provided Seurat object. Subsequently, clustifyr uses a Spearman correlation-based method to find the query cluster expression profile (WT-DAPT clustering resolution parameter 1.0) with the highest similarities. Finally, query clusters were assigned reference cluster labels based on the highest correlation with an automatic cutoff threshold of 0.8 × highest correlation coefficient among the clusters. Using the cluster-to-cell-state assignment available in the metadata from Braun et al. (2022), the transferred “clusters” labels in WT-DAPT were translated to their cell type equivalent (Braun et al. 2022).

### IGV visualization

ChIP-seq and ATAC-seq data sets were visually explored using the Interactive Genomics Viewer (IGV) (Robinson et al. 2011; Thorvaldsdóttir et al. 2013). Images of genomic regions used as proof of principle to demonstrate binding and/or accessibility dynamics within different genotypic conditions were exported as .svg files and cropped with Adobe Illustrator (Adobe).

### Global data visualization

All global visualization of specific ChIP-seq and ATAC-seq data sets (heat maps) was performed using deepTools (Ramírez et al. 2016). The combination of the following two commands was used to generate coverage heat maps that display an average of normalized read density for specific subsets of peaks: computeMatrix reference − point −S (.bigWig) <> −R <.bed> <> n –o <files.gz> −b 1000/3000 −a 1000/3000 −−referencePoint center and plotHeatmap –m <files.gz> −o <Heatmap.svg> n −−colorMap # −−heatmapHeight # −−heatmapWidth #.

### Activity by contact (ABC) algorithm

Enhancer–gene connections were established using the activity by contact (ABC) model (Fulco et al. 2019) for the wild-type condition using the information obtained from the ATAC-seq (MACS “–narrow_peak” calling mode), the H3K27ac ChIP-seq (MACS “–broad” peak calling mode), and RNA-seq in wild-type DIV24 cultures. Each data type was analyzed as specified above. ABC scores for each gene and chromatin-accessible element within a 5-Mb range were calculated. To generate the necessary gene and TSS annotation files, we used the GRCh37.75 annotation in R-3.6.2 ([R Core Team 2019) and the Bioconductor package plyranges (version 1.14.0) (Lee et al. 2019). Transcription start sites (TSSs) for each gene were selected based on the most highly expressed isoform (highest mean TPM expression across the three replicates in the RNA-seq). In cases in which several isoforms showed equal expression levels, we selected the TSS that was used by the majority of isoforms. Last, for the remaining genes (i.e., those for which neither gene expression nor the majority vote identified a unique TSS), we selected the most 5′ TSS. The TSS region was then defined as the 500 bp surrounding each gene's TSSs. We removed genes corresponding to small RNAs (gene symbol contains “MIR” or “RNU,” genes with a gene body length <300 bp; we calculated the gene body length by summing across the exon widths of each transcript). For the gene annotation, each gene was collapsed to its most expanded genomic range.

#### Defining candidate elements

Instead of the makeCandidateRegions.py script, we used the Bioconductor package DiffBind (Ross-Innes et al. 2012). We ran MACS (Zhang et al. 2008) for each ATAC-seq replicate using the ABC algorithm-specific parameters (-p 0.1 –call-summits TRUE) and removed elements overlapping regions of the genome that had been observed to accumulate anomalous number of reads in epigenetic sequencing available via the ENCODE project (ENCODE Project Consortium; Luo et al. 2020) for GRCh37 with the following identifier: ENCSR636HFF. Subsequently, reads were counted with DiffBind::dba.count(DBA, summits = 275, minOverlap = 2) in the consensus peaks identified with DiffBind. Peaks in the consensus peak set were recentered and trimmed based on their points of greatest read overlap (summits) to provide more standardized peak intervals. After background normalization, candidate putative enhancer regions were identified as those 150,000 consensus peaks with the highest mean normalized read count. Finally, we merged the candidate putative enhancer regions with the annotated TSS file region (“include-list”), as the ABC model considers promoters as part of the putative enhancer set.

#### Quantifying enhancer activity

The activity of the putative enhancer regions was then quantified using the run.neighborhoods.py function from the ABC algorithm, including the information for the RNA sequencing to define expressed genes.

#### Computing the ABC score

Finally, ABC scores were calculated using the predict.py without experimental contact data information (using the following parameters: –score_column powerlaw.Score –threshold .022 –make_all_putative).

### Statistics

Where data are presented as the mean ± standard error of the mean (SEM), unpaired Student's *t*-test was used to determine statistical significance (GraphPad Prism and R). Details of statistical analyses are in the figure legends.

### Data availability

All scRNA-seq, RNA-seq, ATAC-seq, and ChIP-seq data sets generated in this study are available for download at GSE214383.

The protein interactions from this study have been submitted to the IMEx (http://www.imexconsortium.org) consortium through IntAct and assigned the identifier IM-29616.

The code and software used are available in Supplemental Table S4.

## Supplementary Material

Supplemental Material
