# Supplementary material for: Pioneer factor ASCL1 cooperates with the mSWI/SNF complex at distal regulatory elements to regulate human neural differentiation
Source: Genes Dev. 2023 Mar 1;37(5-6):218–42. doi: 10.1101/gad.350269.122 (PMC10111863; doi:10.1101/gad.350269.122)
Supplement: Supplemental Material [file supp_37_5-6_218__DC1.html]

Pioneer factor ASCL1 cooperates with the mSWI/SNF complex at distal regulatory elements to regulate human neural differentiation — Pioneer factor ASCL1 cooperates with the mSWI/SNF complex at distal regulatory elements to regulate human neural differentiation — Supplemental Material 

# Pioneer factor ASCL1 cooperates with the mSWI/SNF complex at distal regulatory elements to regulate human neural differentiation

## Supplemental Material

- Supplemental\_Paun350269\_FigS1.pdf
- Supplemental\_Paun350269\_FigS2.pdf
- Supplemental\_Paun350269\_FigS3.pdf
- Supplemental\_Paun350269\_FigS4.pdf
- Supplemental\_Paun350269\_FigS5.pdf
- Supplemental\_Paun350269\_Tables.pdf
- Supplemental\_Paun350269\_FileS1.xlsx
- Supplemental\_Paun350269\_FileS2.txt
- Supplemental\_Paun350269\_FileS3.xlsx
- Supplemental\_Paun350269\_FileS4.xlsx
- Supplemental\_Paun350269\_FileS5.xlsx
