# Supplementary material for: Pioneer factor ASCL1 cooperates with the mSWI/SNF complex at distal regulatory elements to regulate human neural differentiation
Source: Genes Dev. 2023 Mar 1;37(5-6):218–42. doi: 10.1101/gad.350269.122 (PMC10111863; doi:10.1101/gad.350269.122)
Supplement: Supplemental Material [file supp_gad.350269.122_Supplemental_Paun350269_FigS1.pdf]

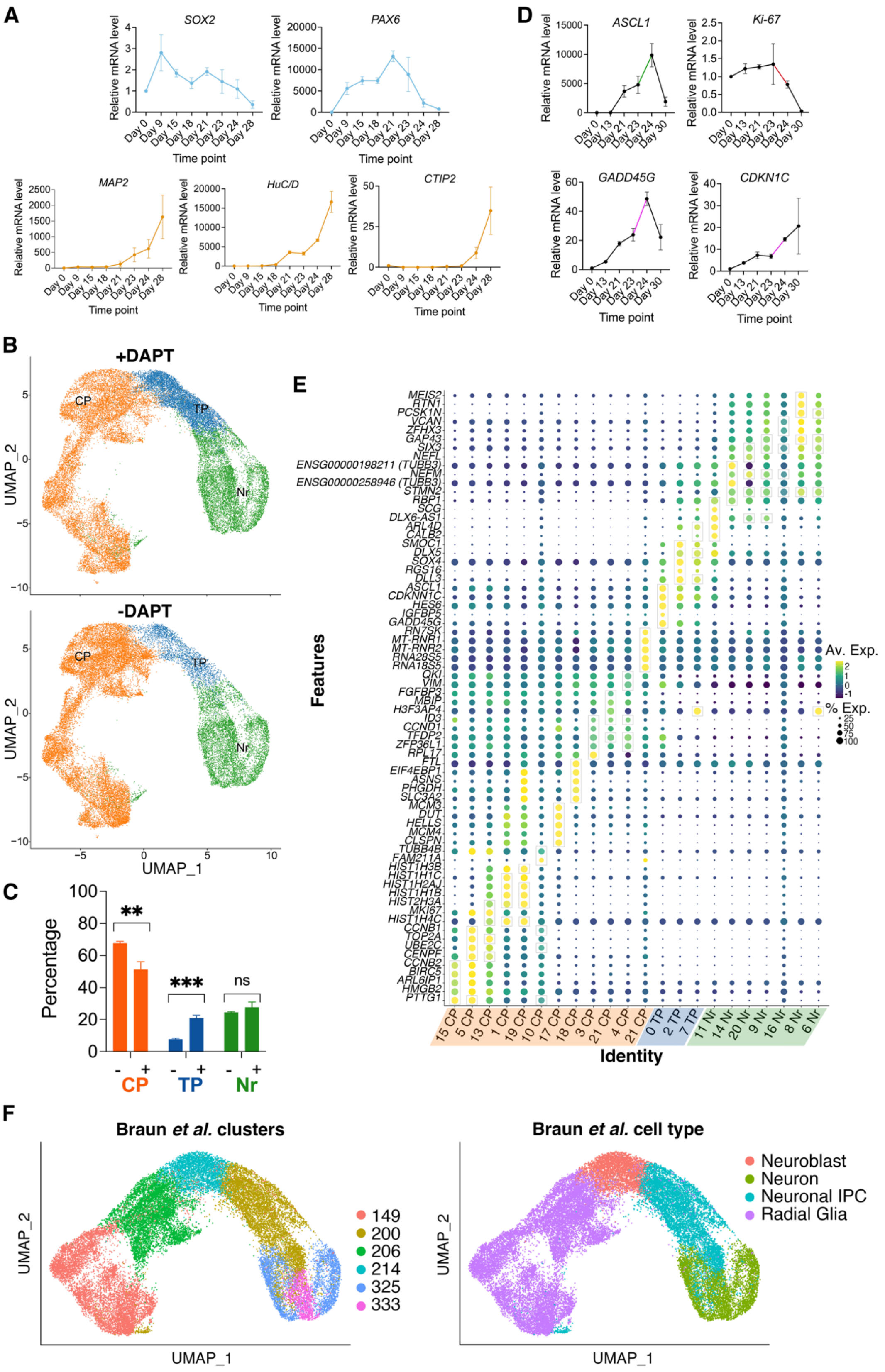

Figure S1, related to Figure 1. ASCL1 expression marks a transitional cell population bridging actively dividing progenitors and postmitotic neurons. (A) qRT-PCR analysis of the expression of neural progenitor-associated genes *SOX2* and *PAX6* (top) activated after

neural induction with dual SMAD inhibition; mRNA expression relative to DIV0. Onset of expression of neuronal genes *MAP2*, *HUC/D* (*ELAVL3/4*) and *CTIP2* (*BCL11B*) is between DIV18 and 21, with marked increase from DIV24 (post Notch inhibition with DAPT) (bottom). Data from same experiment as Figure 1A (three independent cultures). Error bars represent mean ±SEM for three biological replicates. **(B)** UMAP plot projections of single-cell transcriptomes of DIV24 neural cultures, collected from cultures both with and without Notch inhibition at DIV23; a UMAP embedding was estimated after integration of datasets (three replicates in each condition) and then cell types were assigned using the markers shown in Figure 1F; different experiments with (+DAPT) and without (-DAPT) Notch inhibition are shown on the integrated UMAP. Dots represent single cells. Colors represent the different clusters defined by marker expression. CP, cycling progenitors; Nr, neurons; TP, Transitional Progenitors. **(C)** Relative proportion of different cell state clusters from each dataset from (B) showing significant enrichment of Transitional Progenitors with DAPT addition. Unpaired t-test, $^{**}p<0.01$, $^{***}p<0.001$. **(D)** qRT-PCR analysis of the expression of *ASCL1*, *MKI67* (cell proliferation marker) and *CDKN1C* and *GADD45G* (cell cycle exit markers) at multiple timepoints during neural differentiation; mRNA expression relative to DIV0. Colored lines highlight expression changes upon addition of Notch inhibitor DAPT: the increase in *ASCL1* expression between DIV23 and DIV24 is accompanied by a decrease in *MKI67* and increase in *GADD45G* AND *CDKN1C*. Data obtained from an independent experiment from Figure 1A and panel (A). Error bars represent mean ±SEM for three biological replicates. **(E)** Dot plot representation of the expression of all the top 5 genes enriched in the 22 clusters identified by Seurat in Figure 1C (the top 5 for each cluster are outlined in boxes) in comparison to the rest of the cells in the dataset. Dot size indicates percentage of cells in each cluster expressing a gene, shading indicates the average gene expression. **(F)** UMAP projection of DIV24 WT DAPT treated neural cultures with labels transferred from Braun et al., 2022. Human fetal forebrain scRNA-seq cluster transfer shown on the left, and their respective cell types shown on the right (see Fig. 1 for reference). Left, colours indicate cluster number with highest correlation score with current dataset clusters; correlation coefficients are listed in Supplementary Table S1. Right, colors indicate cell type assigned by Braun *et al.* to clusters shown on the left.
