# Supplementary material for: Pioneer factor ASCL1 cooperates with the mSWI/SNF complex at distal regulatory elements to regulate human neural differentiation
Source: Genes Dev. 2023 Mar 1;37(5-6):218–42. doi: 10.1101/gad.350269.122 (PMC10111863; doi:10.1101/gad.350269.122)
Supplement: Supplemental Material [file supp_gad.350269.122_Supplemental_Paun350269_FigS2.pdf]

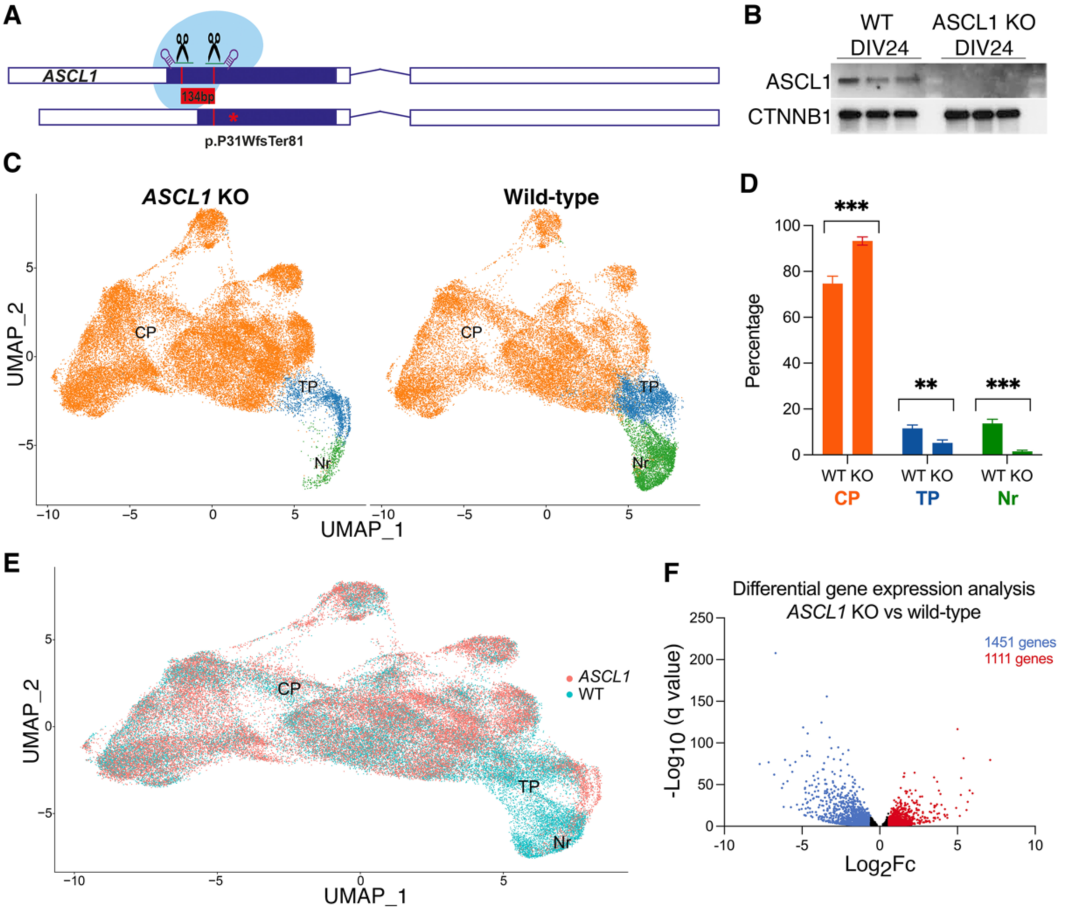

Figure S2, related to Figure 3. Generation of Transitional Progenitors and Neurons is impaired in *ASCL1* KO DIV24 cultures. (A) Diagram illustrating the 133bp deletion in the first exon of *ASCL1* generated by CRISPR/Cas9 (gRNA site targeting represented by scissors), inducing a frameshift with a premature stop codon (represented by a red asterisk), expected to undergo nonsense mediated decay. **(B)** Western blot analysis of ASCL1 protein expression in DIV24 neural cultures of three control (wild-type) and three *ASCL1* KO clones showing no protein expression (after 10 minutes exposure to chemiluminescent reagent). CTNNB1 loading control is included. **(C)** UMAP representations illustrating the three main cell populations (CP, cycling progenitors; Nr, neurons; TP, Transitional Progenitors). *ASCL1* KO cells (left) and wild-type cells (right) are shown as UMAP plot for the integrated datasets from both conditions. **(D)** Proportion of cells in each of the three main populations (CP, Cycling Progenitors; TP, Transitional Progenitors; Nr, Neurons) in wild-type and *ASCL1* KO DIV24 neural cultures (derived from (C)). Unpaired t-test; $^{**}p<0.01$, $^{***}p<0.001$. **(E)** UMAP from (C) with cells grouped by genotype, showing that wild-type and *ASCL1* KO transitional progenitors and neurons cluster separately, suggesting transcriptome-wide differences in those cell types. **(F)** Volcano plot showing the genes dysregulated in *ASCL1* KO versus wild-type DIV24 cultures (bulk RNA-seq); genes with significant (fold change > 1.5, q-value < 0.05) differential gene expression are colored blue (downregulated) and red (upregulated).
