# Supplementary material for: Pioneer factor ASCL1 cooperates with the mSWI/SNF complex at distal regulatory elements to regulate human neural differentiation
Source: Genes Dev. 2023 Mar 1;37(5-6):218–42. doi: 10.1101/gad.350269.122 (PMC10111863; doi:10.1101/gad.350269.122)
Supplement: Supplemental Material [file supp_gad.350269.122_Supplemental_Paun350269_FigS3.pdf]

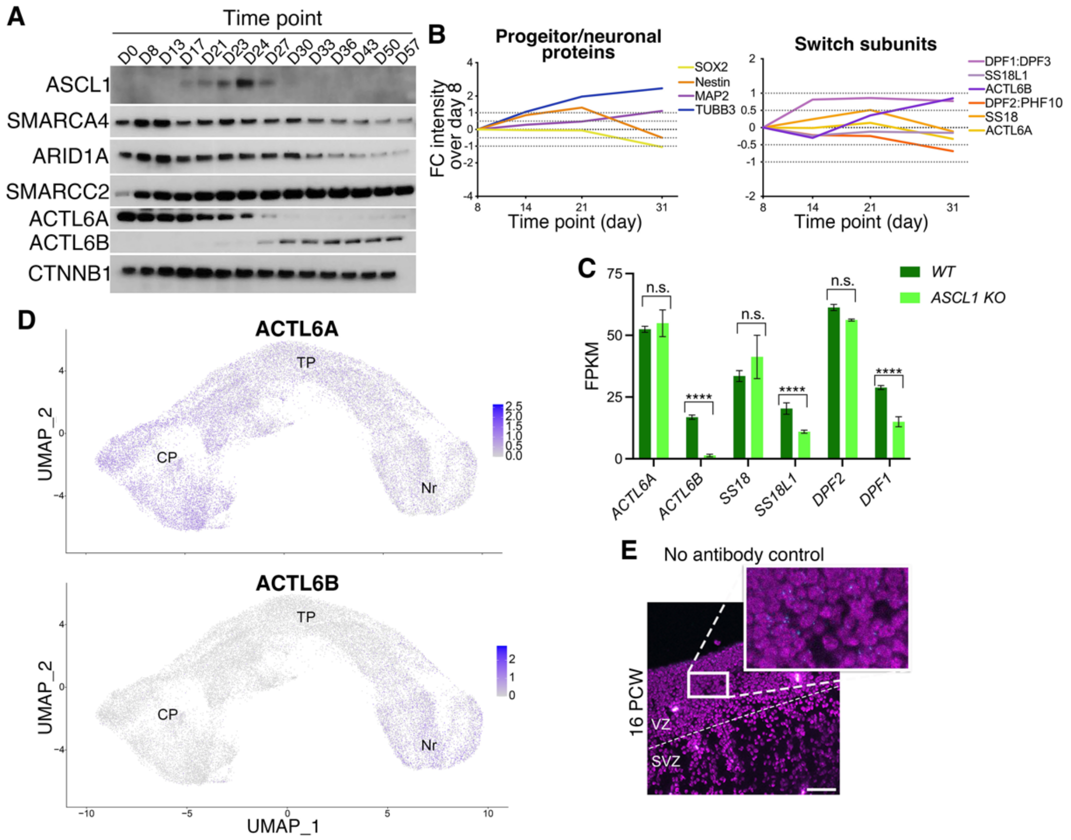

Figure S3, related to Figure 5. mSWI/SNF npBAF and nBAF subunits in human iPSC-derived neural cultures. (A) Western blot showing the expression patterns of ASCL1, mSWI/SNF core and switching subunits. CTNNB1 loading control is included. **(B)** Time course plots of proteins detected using TMT-labelled LC-MS/MS quantitation showing the decrease in expression of npBAF subunits DPF2, PHF10, ACTL6A during differentiation of neural cultures, parallel to the decrease in expression of the progenitor-specific markers SOX2 and Nestin. Conversely, nBAF subunits DPF1/DPF3, SS18L1, ACTL6B increase in expression, in parallel with the expression of the neuronal-specific markers MAP2 and TUBB3. **(C)** Normalized FPKM from RNA-Seq analysis for npBAF and nBAF subunits in wild-type and *ASCL1* KO DIV24 cultures. There is no change in the expression of npBAF subunits *ACTL6A*, *SS18*, or *DPF2*, while all nBAF subunits (*ACTL6B*, *SS18L1*, *DPF1*) are significantly downregulated. ****$padj$<0.0001. **(D)** UMAP plots showing the expression of *ACTL6A* and *ACTL6B* in control cultures at DIV24; single cell gene expression is overlayed on the UMAP from Figure 1D. Transitional progenitors do not express nBAF subunit ACTL6B. CP, Cycling Progenitors; TP, Transitional Progenitors; Nr, Neurons. **(E)** Representative immunofluorescence image of Proximity Ligation Assay no antibody control (related to Figure 5B) in the human fetal cortex at 16 PCW. Scale bar, 50um.
