# Supplementary material for: Pioneer factor ASCL1 cooperates with the mSWI/SNF complex at distal regulatory elements to regulate human neural differentiation
Source: Genes Dev. 2023 Mar 1;37(5-6):218–42. doi: 10.1101/gad.350269.122 (PMC10111863; doi:10.1101/gad.350269.122)
Supplement: Supplemental Material [file supp_gad.350269.122_Supplemental_Paun350269_FigS4.pdf]

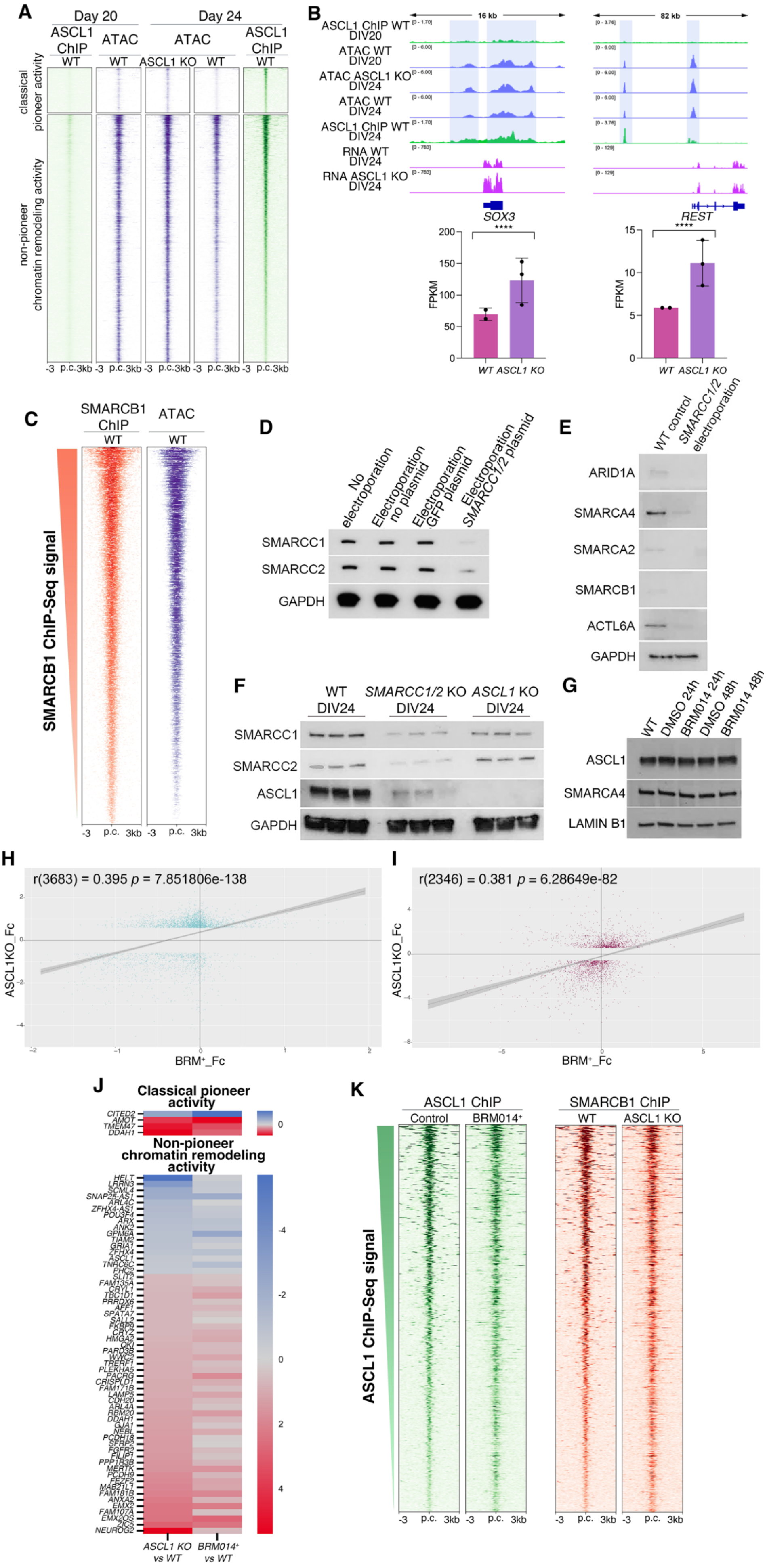
A
Day 20
Day 24
ASCL1 ChIP
ATAC
ATAC
ASCL1 ChIP
WT
WT
ASCL1 KO
WT
WT
classical pioneer activity
non-pioneer chromatin remodeling activity
B
ASCL1 ChIP WT DIV20
ATAC WT DIV20
ATAC ASCL1 KO DIV24
ATAC WT DIV24
ASCL1 ChIP WT DIV24
RNA WT DIV24
RNA ASCL1 KO DIV24
16 kb
82 kb
SOX3
REST
FPKM
WT
ASCL1 KO
C
SMARCB1 ChIP
ATAC
SMARCB1 ChIP-Seq signal
D
No electroporation
Electroporation no plasmid
Electroporation GFP plasmid
Electroporation SMARCC1/2 plasmid
SMARCC1
SMARCC2
GAPDH
E
WT control
SMARCC1/2 electroporation
ARID1A
SMARCA4
SMARCA2
SMARCB1
ACTL6A
GAPDH
F
WT DIV24
SMARCC1/2 KO DIV24
ASCL1 KO DIV24
SMARCC1
SMARCC2
ASCL1
GAPDH
G
WT
DMSO 24h
BRM014 24h
DMSO 48h
BRM014 48h
ASCL1
SMARCA4
LAMIN B1
H
r(3683) = 0.395 p = 7.851806e-138
ASCL1KO_Fc
BRM+_Fc
I
r(2346) = 0.381 p = 6.28649e-82
ASCL1KO_Fc
BRM+_Fc
J
Classical pioneer activity
Non-pioneer chromatin remodeling activity
ASCL1 KO vs WT
BRM014+ vs WT
K
ASCL1 ChIP
Control
BRM014+
SMARCB1 ChIP
WT
ASCL1 KO
ASCL1 ChIP-Seq signal

Figure S4, related to Figures 4 and 6. Interference with mSWI/SNF ATPase activity confirms co-dependency of ASCL1 and mSWI/SNF at a subset of regulatory elements. **(A)** Heatmaps representing the analysis of temporal dynamics of ASCL1 binding and chromatin accessibility that divides its pioneer activity into classical pioneer (top, n= 760) and non-pioneer chromatin remodeling (bottom, n= 4,108) activities at sites where ASCL1 represses accessibility. **(B)** Representative IGV tracks of relevant loci identified in (A). Bottom, bar plots show mean expression in FPKM for the depicted genes in wild-type and *ASCL1*-KO. ****$padj$ <0.0001. **(C)** Heatmaps representing SMARCB1 binding by ChIP-Seq (n=26,207) relative to chromatin state determined by ATAC-Seq in wild type DIV24 cultures. **(D)** Western blot for SMARCC1 and SMARCC2 on the collection day (DIV24) for cells that underwent electroporation with the plasmids targeting *SMARCC1* and *SMARCC2* and their respective controls. GAPDH loading control is included. **(E)** Western blot showing the protein levels of other mSWI/SNF subunits (ARID1A, SMARCA2, SMARCA4, SMARCB1, ACTL6A) in the SMARCC1/2 KO cells compared to control cells at DIV24. GAPDH loading control is included. **(F)** Western blot analysis of SMARCC1, SMARCC2 and ASCL1 expression in wild-type, *SMARCC1/2* KO and *ASCL1* KO DIV24 cultures. GAPDH loading control is included. **(G)** Western blot analysis of ASCL1 and SMARCA4 expression at 24 and 48h after treatment with ATPase inhibitor BRM014 (and vehicle control) showing treatment does not affect protein expression. LaminB1 loading control is included. **(H)** Scatterplot showing the change in accessibility assessed by ATAC-Seq in BRM014-treated cells versus control cultures at all ASCL1-bound sites that show a significant change in accessibility upon ASCL1 removal. Blue dots represent sites that also reach the significance threshold after 48hrs of BRM014 inhibitory treatment. Red dots represent sites that do not reach the significance threshold after 48hrs of BRM014 inhibitory treatment. Fc: fold change. **(I)** Scatterplot showing the changes at the transcriptional level assessed by RNA-Seq in BRM014-treated cells versus control cultures at all genes found to be significantly dysregulated upon ASCL1 removal. Blue dots represent genes that also reach the significance threshold after 48hrs of BRM014 inhibitory treatment (BRM sig). Fc: fold change. **(J)** Heatmaps showing the ABC-predicted genes dysregulated in both *ASCL1* KO and BRM014-treated cultures where ASCL1-SMARCB1 co-binding induces decreased accessibility at ASCL1 classical pioneer activity sites (top) and sites of ASCL1 non-pioneer chromatin remodeling activity (bottom). **(K)** Heatmaps profiling ASCL1 and SMARCB1 binding at ASCL1-mSWI/SNF dependent sites where the interaction is associated with closed chromatin, showing that interfering with mSWI/SNF ATPase activity (BRM014 treatment) reduces ASCL1 binding and reciprocally, eliminating ASCL1 (*ASCL1* KO) reduces SMARCB1 binding.
