# Supplementary material for: Pioneer factor ASCL1 cooperates with the mSWI/SNF complex at distal regulatory elements to regulate human neural differentiation
Source: Genes Dev. 2023 Mar 1;37(5-6):218–42. doi: 10.1101/gad.350269.122 (PMC10111863; doi:10.1101/gad.350269.122)
Supplement: Supplemental Material [file supp_gad.350269.122_Supplemental_Paun350269_FigS5.pdf]

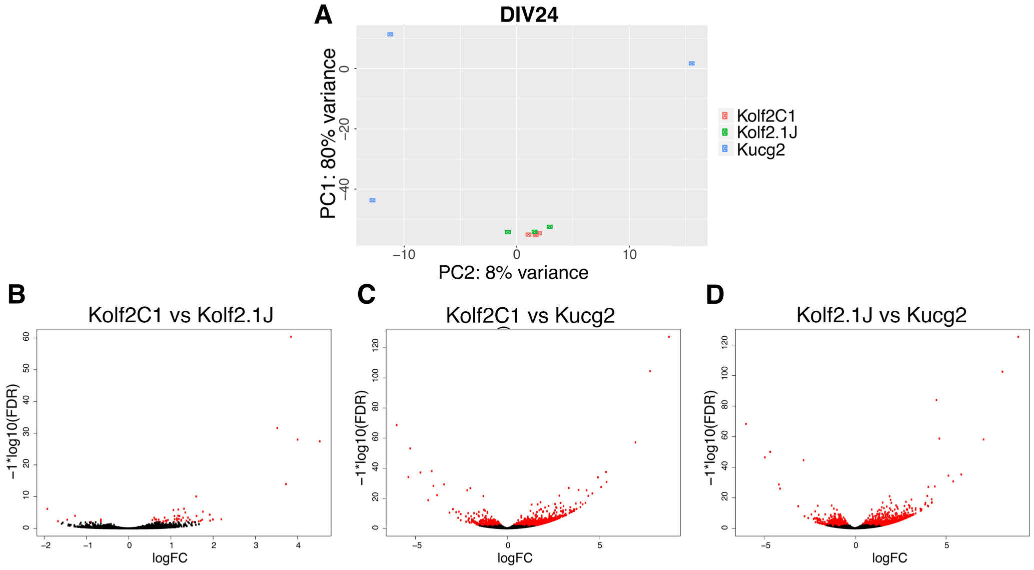

Figure S5, related to Methods. Differential transcriptomic analysis of DIV24 neuronal cultures derived from three different iPSC lines. (A) PCA scatter plot of gene expression in three iPSC-line-derived neuronal cultures at DIV24: Kolf2C1, Kolf2.1J (CRISPR/Cas9 edited Kolf2C1) and independent line Kucg2. **(B, C, D)** Volcano plots representing differential gene expression analysis between pairs of the three lines tested in (A). Red dots indicate q<0.05.
