# Supplementary material for: Pioneer factor ASCL1 cooperates with the mSWI/SNF complex at distal regulatory elements to regulate human neural differentiation
Source: Genes Dev. 2023 Mar 1;37(5-6):218–42. doi: 10.1101/gad.350269.122 (PMC10111863; doi:10.1101/gad.350269.122)
Supplement: Supplemental Material [file supp_gad.350269.122_Supplemental_Paun350269_Tables.pdf]

Supplemental information

Supplemental Tables

Supplemental Table S1. Correlation coefficient for the label transfer from Braun et. al. to DIV24 WT DAPT.

Supplemental Table S2. Media and buffer recipes.

Supplemental Table S3. List of qRT-PCR TaqMan probes.

Supplemental Table S4. Resources Table

Supplemental Table S5. ATAC-seq primers with barcodes (Buenrostro et al., 2013).

Supplemental Table S6. List of genes used to group DIV24 cells into three populations: cycling progenitors, transitional progenitors, and neurons.

Supplemental Table S7. Samples subsetted from Braun *et. al.* for label transfer to DIV24 WT DAPT (see Suppl. Fig. S1F)

Supplemental Figures

Supplemental Figure S1, related to Figure 1. ASCL1 expression marks a transitional cell population bridging actively dividing progenitors and postmitotic neurons.

Supplemental Figure S2, related to Figure 3. Generation of Transitional Progenitors and Neurons is impaired in ASCL1 KO DIV24 cultures.

Supplemental Figure S3, related to Figure 5. mSWI/SNF npBAF and nBAF subunits in human iPSC-derived neural cultures.

Supplemental Figure S4, related to Figures 4 and 6. Interference with mSWI/SNF ATPase activity confirms co-dependency of ASCL1 and mSWI/SNF at a subset of regulatory elements.

Supplemental Figure S5, related to Methods. Differential transcriptomic analysis of DIV24 neuronal cultures derived from three different iPSC lines.

Supplemental Files

Supplemental File S1. Differential gene expression analysis of *ASCL1* KO vs control neural cultures at DIV24.

Supplemental File S2. Distal regulatory element map in DIV24 wild type neural cultures predicted by the ABC algorithm (annotated to GRCh37).

Supplemental File S3. Related to Fig. 3E,F. ASCL1-regulated genes: differential gene expression and Reactome pathway and gene ontology analyses.

Supplemental File S4. Motifs from HOMER “known motif" analysis for different categories of ASCL1 and SMARCB1-bound sites.

Supplemental File S5. Reactome pathway and gene ontology overrepresentation analyses of ABC predicted genes co-regulated ASCL1 and mSWI/SNF

Supplemental Tables

Supplemental Table S1. Correlation coefficient for the label transfer from Braun *et. al.* to DIV24 WT DAPT (see Suppl. Fig. S1F)

WT DAPT cluster_res.1	WT DAPT Cell state	Clustifyr correlation	Braun *et al.* clusters	Braun *et al.* cell class
0	Transitional Progenitors	0.7617592	214	Neuroblast
2	Transitional Progenitors	0.77240145	200	Neuronal IPC
7	Transitional Progenitors	0.78941559	200	Neuronal IPC
1	Cycling Progenitors	0.83877441	149	Radial glia
13	Cycling Progenitors	0.85538751	149	Radial glia
10	Cycling Progenitors	0.81792594	149	Radial glia
5	Cycling Progenitors	0.85414473	149	Radial glia
19	Cycling Progenitors	0.81907415	149	Radial glia
3	Cycling Progenitors	0.80117349	206	Radial glia
4	Cycling Progenitors	0.80137705	206	Radial glia
18	Cycling Progenitors	0.76907844	206	Radial glia
17	Cycling Progenitors	0.81099399	206	Radial glia
12	Cycling Progenitors	0.80609632	206	Radial glia
15	Cycling Progenitors	0.83524012	149	Radial glia
21	Cycling Progenitors	0.80200908	206	Radial glia
16	Neurons	0.77417667	200	Neuronal IPC
8	Neurons	0.80739526	333	Neuron
20	Neurons	0.78044896	325	Neuron
9	Neurons	0.80706875	325	Neuron
11	Neurons	0.80906748	200	Neuronal IPC
14	Neurons	0.78092349	200	Neuronal IPC
6	Neurons	0.79754045	325	Neuron

Supplemental Table S2. Media and Buffer Recipes

Component	Stock Concentration	Final Concentration	Volume
**N2 media composition (500 ml)**			
DMEM/F-12	-	97%	485 ml
N-2 Supplement	100 X	1 X	5 ml
GlutaMAX-I Supplement	100 X	1 X	5 ml
Penicillin/Streptomycin	10,000 μg/ml (100X)	100 μg/ml	5 ml
**B27 media composition (500 ml)**			
Neurobasal Medium	-	96%	480 ml
B-27 Supplement	50 X	1 X	10 ml
GlutaMAX-I Supplement	100 X	1%	5 ml
Penicillin/Streptomycin	10,000 μg/ml (100X)	100 μg/ml	5 ml
**ChIP SDS Lysis Buffer (50 ml)**			
Tris-HCl, pH 7.5	1M	50mM	2.5 ml
EDTA	0.5M	10mM	1 ml
SDS	20%	1%	2.5 ml
Water	-	-	44 ml
**ChIP Chromatin Dilution Buffer (50 ml)**			
Tris, pH 7.5	1M	25mM	1.25 ml

Component	Stock Concentration	Final Concentration	Volume
EDTA	0.5M	5mM	0.5 ml
Triton X-100	100%	1%	0.5 ml
SDS	20%	0.1%	0.25 ml
Water	-	-	47.5 ml
**ChIP Wash Buffer A (50 ml)**			
HEPES, pH 7.9	0.5M	50mM	5 ml
NaCl	5M	140mM	1.4 ml
EDTA	0.5M	1mM	0.1 ml
Triton X-100	100%	1%	0.5 ml
Sodium deoxycholate	10%	0.1%	0.5 ml
SDS	20%	0.1%	0.25 ml
Water	-	-	42.25 ml
**ChIP Wash Buffer B (50 ml)**			
HEPES, pH 7.9	0.5M	50mM	5 ml
NaCl	5M	500mM	5 ml
EDTA	0.5M	1mM	0.1 ml
Triton X-100	100%	1%	0.5 ml
Sodium deoxycholate	10%	0.1%	0.5 ml
SDS	20%	0.1%	0.25 ml
Water	-	-	38.65 ml
**Component**	**Stock Concentration**	**Final Concentration**	**Volume**
**ChIP Wash Buffer C (50 ml)**			
Tris, pH 8.0	1M	20mM	1 ml
EDTA	0.5M	1mM	0.1 ml
LiCl	8M	250mM	1.56 ml
NP-40 Alternative	100%	0.5%	0.25 ml
Sodium deoxycholate	10%	0.5%	2.5 ml
Water	-	-	44.59 ml
**ChIP TE Buffer (50 ml)**			
Tris, pH 8.0	1M	10mM	0.5 ml
EDTA	0.5M	1mM	0.1 ml
Water	-	-	49.4 ml
**ChIP Elution Buffer (50 ml)**			
Tris, pH 7.5	1M	10mM	0.5 ml
EDTA	0.5M	1mM	0.1 ml
SDS	20%	1%	2.5 ml
Water	-	-	46.9 ml
**ATAC RSB Buffer (50 ml)**			
Tris-HCl, pH 7.4	1M	10mM	0.5 ml
NaCl	5M	10mM	0.1mM
$MgCl_2$	1M	3mM	0.15 ml
Water	-	-	49.25 ml

Supplemental Table S3. List of qRT-PCR TaqMan® probes

Target Gene	Supplier	Assay ID	Application
ASCL1	ThermoFisher Scientific	Hs00269932_m1	qRT-PCR
BCL11B/CTIP2	ThermoFisher Scientific	Hs01102259_m1	qRT-PCR
CDK1C	ThermoFisher Scientific	Hs00175938_m1	qRT-PCR
GADD45G	ThermoFisher Scientific	Hs00198672_m1	qRT-PCR
HPRT1	ThermoFisher Scientific	Hs02800695_m1	qRT-PCR
HuC/D	ThermoFisher Scientific	Hs00956610_mH	qRT-PCR
MAP2	ThermoFisher Scientific	Hs00258900_m1	qRT-PCR
MKI67	ThermoFisher Scientific	Hs00606991_m1	qRT-PCR
PAX6	ThermoFisher Scientific	Hs00240871_m1	qRT-PCR
SOX2	ThermoFisher Scientific	Hs01053049_s1	qRT-PCR
UBC	ThermoFisher Scientific	Hs00824723_m1	qRT-PCR

Supplemental Table S4. Resources Table

REAGENT or RESOURCE	SOURCE	IDENTIFIER
**Antibodies**		
Rabbit monoclonal anti-ACTL6A (IF, WB)	Abcam	Cat# ab131272; RRID: AB_11157110
Rabbit monoclonal anti-ACTL6B (IF, PLA, WB)	Abcam	Cat# ab180927 RRID: AB_2924269
Rabbit polyclonal anti-ARID1A (IP, WB)	Bethyl	Cat# A301-041A RRID: AB_2060365
Mouse monoclonal anti-ASCL1 (IF, IP, WB)	BD Biosciences	Cat# 556604 RRID: AB_396479
Rabbit monoclonal anti-ASCL1 (IF, FACS, PLA, WB)	Abcam	Cat# ab211327 RRID: AB_2924270
Rabbit polyclonal anti-ASCL1 (ChIP)	Abcam	Cat# ab74065 RRID: AB_1859937
Mouse anti-CTNNB1 (WB)	BD Biosciences	Cat# 610154 RRID: AB_397555
Rat monoclonal anti-CTIP2 (IF)	Abcam	Cat# ab18465 RRID: AB_2064130
Mouse monoclonal anti-GAPDH (WB)	Santa Cruz	Cat# sc-47724 RRID: AB_627678
Rabbit monoclonal anti-GAPDH (WB)	Cell Signaling Technology	Cat# 5174 RRID: AB_10622025
Rabbit polyclonal anti-PAX6 (IF)	Covance	Cat# PRB-278P RRID: AB_291612
Rabbit polyclonal anti-SMARCA4 (WB)	Santa Cruz	Cat# sc-10768 RRID: AB_2255022
Mouse monoclonal anti-SMARCB1 (PLA, WB)	BD Biosciences	Cat# 612110 RRID: AB_399481
Rabbit polyclonal anti-SMARCB1 (ChIP)	Abcam	Cat# ab12167 RRID: AB_298898
Rabbit polyclonal anti-SMARCC1 (IP, WB)	Abcam	Cat# ab72503 RRID: AB_1270780
Rabbit polyclonal anti-SMARCC2 (PLA, WB)	Bethyl	Cat# A301-038A
Rat monoclonal anti-SOX2 (FACS)	eBioscience	Cat# 14-9811-82 RRID: AB_2924272
Mouse monoclonal anti-TUBB3 (FACS)	Covance	Cat# MMS-435P RRID: AB_2313773
Normal Rabbit IgG (IP)	Cell Signaling Technology	Cat# 2729 RRID: AB_1031062
Normal Rabbit IgG (IP)	Cell Signaling Technology	Cat# 3900 RRID: AB_1550038
Normal Mouse IgG (IP)	Cell Signaling Technology	Cat# 5415 RRID: AB_10829607
Alexa Fluor donkey anti-rabbit IgG (IF)	Invitrogen	Cat# A21206 (488) RRID: AB_2535792 Cat# A21207 (594) RRID: AB_141637
Alexa Fluor donkey anti-rabbit IgG (IF)	Jackson ImmunoResearch	Cat# 711-606-152 RRID: AB_2340625

REAGENT or RESOURCE	SOURCE	IDENTIFIER
Alexa Fluor donkey anti-mouse IgG (IF)	Invitrogen	Cat# A21202 (488)<br>RRID: AB_141607<br>Cat# A21203 (594)<br>RRID: AB_141633
Alexa Fluor donkey anti-mouse IgG (IF)	Jackson ImmunoResearch	Cat# 715-606-151<br>RRID: AB_2340866
Alexa Fluor donkey anti-goat IgG (IF)	Invitrogen	Cat# A21447 (647)<br>RRID: AB_2535864
Alexa Fluor donkey anti-rat IgG (IF)	Invitrogen	Cat# A21208 (488)<br>RRID: AB_2535794<br>Cat# A21209 (594)<br>RRID: AB_2535795
Rabbit Anti-Mouse Immunoglobulins HRP (WB)	Dako	Cat# P0161<br>RRID: AB_2687969
Goat Anti-Rabbit Immunoglobulins HRP (WB)	Dako	Cat# P0448<br>RRID: AB_2617138
Anti-Rabbit IgG HRP (WB post IP)	Rockland	Cat# 18-8816-31<br>RRID: AB_2610847
Anti-Mouse Ig HRP (WB post IP)	Rockland	Cat# 18-8817-31<br>RRID: AB_2610850
**Biological samples**		
Human fetal tissue	MRC-Wellcome Trust Human Developmental Biology Resource	http://hdbr.org
**Experimental models: Cell lines**		
clone C1 of parental line HPSI0114ikolf2	Wellcome Sanger Institute	www.hipsci.org
KOLF2.1J	The Jackson Laboratory for Genomic Medicine	www.hipsci.org
HPSI0214i-kucg_2	Wellcome Sanger Institute	www.hipsci.org
**Chemicals, peptides, and recombinant proteins**		
Geltrex	ThermoFisher Scientific	Cat# A1413201
Synthemax	Sigma-Aldrich	Cat# CLS3535
DPBS	ThermoFisher Scientific	Cat# 14190-094
DMEM/F-12	ThermoFisher Scientific	Cat# 11320033
Neurobasal Medium	ThermoFisher Scientific	Cat# 21103049
N-2 Supplement	ThermoFisher Scientific	Cat# 17502001
B-27 Supplement	ThermoFisher Scientific	Cat# 17504044
GlutaMAX-I Supplement	ThermoFisher Scientific	Cat# 35050-038
Penicillin/ Streptomycin	ThermoFisher Scientific	Cat# 15140122
Y-27632 ROCK inhibitor	Tocris	Cat# 1254/10
SB31542	Abcam	Cat# ab120163
LDN193189	StemCell Technologies	Cat# 72147
DAPT	Cambridge Bioscience	Cat# SM15-10
Accutase	Sigma-Aldrich	Cat# A6964
HBSS	ThermoFisher Scientific	Cat# 14170088
tracrRNA	IDT	Cat# 1072533
IDT duplex buffer	IDT	Cat# 11-05-01-12
Alt-R® S.p. HiFi Cas9 Nuclease V3	IDT	Cat# 1081060

REAGENT or RESOURCE	SOURCE	IDENTIFIER
EP enhancer	IDT	Cat# 1075916
CloneR	Stemcell Technologies	Cat# 05888
Pierce IP lysis buffer	ThermoFisher Scientific	Cat# 87787
Halt™ Protease Inhibitor Cocktail	ThermoFisher Scientific	Cat# 87786
Halt™ Protease Inhibitor Cocktail	ThermoFisher Scientific	Cat# 78420
BSA	ThermoFisher Scientific	Cat# 23209
Sample Buffer, Laemmli 2× Concentrate	Sigma	Cat# S3401-10VL
10x Tris/Glycine/SDS	Bio-Rad	Cat# 1610732
10x Tris Buffered Saline	Bio-Rad	Cat# 1706435
Dried Skimmed Milk Powder	Marvel	N/A
ECL detection reagent	Amersham	Cat# RPN2236
Protein G Sepharose®, Fast Flow	Sigma	Cat# P3296
Paraformaldehyde, 4% in PBS	Alfa Aesar	Cat# J61899
Live/Dead™ Fixable Near-IR Dead Cell Stain	Invitrogen	Cat# L34976
Normal donkey serum	Jackson ImmunoResearch	Cat# 017-000-121
DAPI	Sigma	Cat# D9564
Vectashield Antifade Mounting Medium	Vector Laboratories	Cat# H-1000-10
di(N-succimidyl) glutarate	Sigma-Aldrich	Cat# 80424
Pierce™ 16% Formaldehyde (w/v), Methanol-free	ThermoFisher Scientific	Cat# 28908
Glycine	Sigma-Aldrich	Cat# 50046
Tris-HCl, pH 7.5	In house[1]	N/A
Tris, pH 8.0	In house[1]	N/A
EDTA	ThermoFisher Scientific	Cat# 87788
EDTA	Sigma	Cat# E7889
SDS	ThermoFisher Scientific	Cat# AM9820
Triton X-100	Sigma	Cat# T8787
HEPES,<br>pH 7.9	Sigma	Cat# H3375
Sodium deoxycholate	Sigma	Cat# 30970
LiCl	Sigma	Cat# L7026
NP-40 Alternative	Sigma	Cat# 18896
RNase A	ThermoFisher Scientific	Cat# E0531
Proteinase K	ThermoFisher Scientific	Cat# 78437
Tris-HCl, pH 7.4	In house[1]	N/A
$MgCl_2$	In house[1]	N/A
NaCl	Sigma	Cat# S5150
Water	ThermoFisher Scientific	Cat# AM9937
**Critical commercial assays**		
P3 Primary Cell 4D-Nucleofector™ X Kit L	Lonza	Cat# V4XP-3024
RNeasy Micro Kit	Qiagen	Cat# 74004
Maxima First Strand cDNA Synthesis kit with dsDNase	ThermoFisher Scientific	Cat# K1671
Taqman Universal qRT-PCR Master Mix	ThermoFisher Scientific	Cat# 4304437
Pierce BCA Protein Assay Kit	ThermoFisher Scientific	Cat# 23225

REAGENT or RESOURCE	SOURCE	IDENTIFIER
4–15% Mini-PROTEAN® TGX™ Precast Protein Gels	Bio-Rad	Cat# 4561085
Trans-Blot Turbo Mini 0.2 µm PVDF Transfer Packs	Bio-Rad	Cat# 1704156
Hyperfilm™ ECL™	Amersham	Cat# 28-9068-36
Duoluk In Situ Red Started Kit Mouse/Rabbit	Sigma-Aldrich	Cat# DUO92101-1KT
KAPA mRNA polyA HyperPrep Kit	Illumina	Cat# KR1352
Dynabeads Protein G	ThermoFisher Scientific	Cat# 10003D
Dynabeads Protein A	ThermoFisher Scientific	Cat# 10008D
KAPA pure beads	Roche	Cat# 07893271001
Zymo Clean & Concetrator-5 Kit	Zymo Research	Cat# D4014
NEB Ultra II DNA Library Prep Kit for Illumina	New England BioLabs	Cat# E7103
Illumina Tagment DNA TDE1 Enzyme and Buffer Small Kit	Illumina	Cat# 20034197
NEBNext HiFi 2X PCR Master Mix	New England BioLabs	Cat# M0541S
QubitTM dsDNA HS assay	ThermoFisher Scientific	Cat# Q32851
**Oligonucleotides**		
qRT-PCR Taqman Probes	ThermoFisher Scientific	Supplemental Table 2
Primers for indexing ATAC-seq libraries	(Buenrostro et al. 2013)	Supplemental Table 3
**Deposited data**		
Raw and analyzed data	This paper	GEO: GSE214383<br>https://www.ncbi.nlm.nih.gov/geo/query/acc.cgi?acc=GSE214383
Protein interactions	This paper	http://www.imexconsortium.org (Orchard et al. 2013) ID: IM-29616
Fetal brain scRNA-seq – complete processed dataset .h5 object	(Braun et al. 2022)	https://github.com/linnarsson-lab/developing-human-brain/#:~:text=HumanFetalBrainPool.h5<br>Content description at https://github.com/linnarsson-lab/developing-human-brain/
**Reference data**		
Human reference genome NCBI build 37, GRCh37	Genome Reference Consortium	http://www.ncbi.nlm.nih.gov/projects/genome/assembly/grc/human/
**Software and algorithms**		
All data analysis scripts used in this paper	This paper	https://github.com/strohstern/op17_PM21134_ChIP.git<br>https://github.com/FrancisCrickInstitute/SC21030_OP_ASCL1ko<br>https://github.com/FrancisCrickInstitute/op17_adjusted_ABC
Fiji v2.3.0/1.53q	(Schindelin et al. 2012)	http://fiji.sc/
FlowJo v10.8.1	BD Life Sciences	https://www.flowjo.com
GraphPad Prism 9 v9.4.1	GraphPad	https://www.graphpad.com/
Cutadapt v1.9.1	(Martin 2011)	https://github.com/marcelm/cutadapt/
RSEM v1.3.0	(Li and Dewey 2011)	https://github.com/deweylab/RSEM
STAR alignment algorithm v2.5.2a	(Dobin et al. 2013)	https://github.com/alexdobin/STAR
DESeq2 v1.12.3	(Love et al. 2014)	https://bioconductor.org/packages/release/bioc/html/DESeq2.html

REAGENT or RESOURCE	SOURCE	IDENTIFIER
R	R Foundation	https://www.r-project.org/
DAVID Bioinformatics Resources	(Huang da et al. 2009; Sherman et al. 2022)	https://david.ncifcrf.gov/summary.jsp
nf-core/ChIP-seq pipeline v1.1.0	(Ewels et al. 2020)	https://doi.org/10.5281/zenodo.3529400
nf-core/atacseq pipeline v1.1.0	(Ewels et al. 2020)	https://doi.org/10.5281/zenodo.3529420
BEDTools v2.30.0	(Quinlan and Hall 2010)	https://bedtools.readthedocs.io/en/latest/
DeepTools v3.5.0	(Ramírez et al. 2016)	https://deeptools.readthedocs.io/en/develop/
DiffBind v3.4.11	(Ross-Innes et al. 2012)	https://bioconductor.org/packages/release/bioc/html/DiffBind.html
Activity-by-Contact algorithm	(Fulco et al. 2019)	https://github.com/broadinstitute/ABC-Enhancer-Gene-Prediction
Cell Ranger v5.0.0	10X Genomics	https://www.10xgenomics.com
Seurat v4.1.1	(Butler et al. 2018; Stuart et al. 2019; Hao et al. 2021)	https://satijalab.org/seurat/
scVelo v0.2.2	(Bergen et al. 2020)	https://github.com/theislab/scvelo/blob/master/docs/source/index.rst
HOMER v3.1	(Heinz et al. 2010)	http://homer.ucsd.edu/homer/motif/
Clustifyr v1.10.0	(Fu et al. 2020)	https://github.com/rnabioco/clustifyR
AnnData v 0.8.0	(Virshup et al. 2021)	https://github.com/scverse/anndata
Python v3.9.16	Python Software Foundation Copyright © 2001-2023	https://www.python.org/downloads/release/python-3916/
SeuratDisk v0.0.09020	Paul Hoffman	https://github.com/mojaveazure/seurat-disk
Interactive Genomics Viewer (IGV) v2.12.3	(Robinson et al. 2011; Thorvaldsdottir et al. 2013)	https://software.broadinstitute.org/software/igv/
**Other**		
Amaxa 4D Nucleofector	Lonza	Cat# AAF-1002X
Cell strainer-capped tubes	Falcon	Cat# 352235
1.5 ml Picoruptor Microtubes with Caps	Diagenode	Cat# C30010016
Eppendorf® LoBind microcentrifuge tubes	Fisher Scientific	Cat# 022431081
Agilent TapeStation 4200 System	Agilent	Cat# G2991AA
Qubit 3.0 Fluorometer	Life Technologies	Cat# Q33216

[1] = Media preparation STP, The Francis Crick Institute. IF, immunofluorescence. WB, western blot. PLA, proximity ligation assay. ChIP, chromatin immunoprecipitation. IP, immunoprecipitation. FACS, Fluorescence activated cell sorting.

References to Supplemental Table 3

Bergen V, Lange M, Peidli S, Wolf FA, Theis FJ. 2020. Generalizing RNA velocity to transient cell states through dynamical modeling. *Nat Biotechnol* **38**: 1408-1414.

Braun E, Danan-Gotthold M, Borm LE, Vinsland E, Lee KW, Lönnerberg P, Hu L, Li X, He X, Andrusivová Ž et al. 2022. Comprehensive cell atlas of the first-trimester developing human brain. *bioRxiv*: 2022.2010.2024.513487.

Buenrostro JD, Giresi PG, Zaba LC, Chang HY, Greenleaf WJ. 2013. Transposition of native chromatin for fast and sensitive epigenomic profiling of open chromatin, DNA-binding proteins and nucleosome position. *Nat Methods* **10**: 1213-1218.

Butler A, Hoffman P, Smibert P, Papalexi E, Satija R. 2018. Integrating single-cell transcriptomic data across different conditions, technologies, and species. *Nat Biotechnol* **36**: 411-420.
Dobin A, Davis CA, Schlesinger F, Drenkow J, Zaleski C, Jha S, Batut P, Chaisson M, Gingeras TR. 2013. STAR: ultrafast universal RNA-seq aligner. *Bioinformatics* **29**: 15-21.
Ewels PA, Peltzer A, Fillinger S, Patel H, Alneberg J, Wilm A, Garcia MU, Di Tommaso P, Nahnsen S. 2020. The nf-core framework for community-curated bioinformatics pipelines. *Nat Biotechnol* **38**: 276-278.
Fu R, Gillen AE, Sheridan RM, Tian C, Daya M, Hao Y, Hesselberth JR, Riemondy KA. 2020. clustifyr: an R package for automated single-cell RNA sequencing cluster classification. *F1000Res* **9**: 223.
Fulco CP, Nasser J, Jones TR, Munson G, Bergman DT, Subramanian V, Grossman SR, Anyoha R, Doughty BR, Patwardhan TA et al. 2019. Activity-by-contact model of enhancer–promoter regulation from thousands of CRISPR perturbations. *Nature Genetics* **51**.
Hao Y, Hao S, Andersen-Nissen E, Mauck WM, 3rd, Zheng S, Butler A, Lee MJ, Wilk AJ, Darby C, Zager M et al. 2021. Integrated analysis of multimodal single-cell data. *Cell* **184**: 3573-3587 e3529.
Heinz S, Benner C, Spann N, Bertolino E, Lin YC, Laslo P, Cheng JX, Murre C, Singh H, Glass CK. 2010. Simple combinations of lineage-determining transcription factors prime cis-regulatory elements required for macrophage and B cell identities. *Mol Cell* **38**: 576-589.
Huang da W, Sherman BT, Lempicki RA. 2009. Systematic and integrative analysis of large gene lists using DAVID bioinformatics resources. *Nat Protoc* **4**: 44-57.
Li B, Dewey CN. 2011. RSEM: accurate transcript quantification from RNA-Seq data with or without a reference genome. *BMC Bioinformatics* **12**.
Love MI, Huber W, Anders S. 2014. Moderated estimation of fold change and dispersion for RNA-seq data with DESeq2. *Genome Biology* **15**.
Martin M. 2011. Cutadapt removes adapter sequences from high-throughput sequencing reads. *2011* **17**: 3.
Orchard S, Ammari M, Aranda B, Breuza L, Briganti L, Broackes-Carter F, Campbell NH, Chavali G, Chen C, del-Toro N et al. 2013. The MIntAct project—IntAct as a common curation platform for 11 molecular interaction databases. *Nucleic Acids Research* **42**: D358-D363.
Quinlan AR, Hall IM. 2010. BEDTools: a flexible suite of utilities for comparing genomic features. *Bioinformatics* **26**: 841-842.
Ramírez F, Ryan DP, Grüning B, Bhardwaj V, Kilpert F, Richter AS, Heyne S, Dündar F, Manke T. 2016. deepTools2: a next generation web server for deep-sequencing data analysis. *Nucleic acids research* **44**.
Robinson JT, Thorvaldsdottir H, Winckler W, Guttman M, Lander ES, Getz G, Mesirov JP. 2011. Integrative genomics viewer. *Nat Biotechnol* **29**: 24-26.
Ross-Innes CS, Stark R, Teschendorff AE, Holmes KA, Ali HR, Dunning MJ, Brown GD, Gojis O, Ellis IO, Green AR et al. 2012. Differential oestrogen receptor binding is associated with clinical outcome in breast cancer. *Nature* **481**: 389-393.
Schindelin J, Arganda-Carreras I, Frise E, Kaynig V, Longair M, Pietzsch T, Preibisch S, Rueden C, Saalfeld S, Schmid B et al. 2012. Fiji: an open-source platform for biological-image analysis. *Nat Methods* **9**: 676-682.
Sherman BT, Hao M, Qiu J, Jiao X, Baseler MW, Lane HC, Imamichi T, Chang W. 2022. DAVID: a web server for functional enrichment analysis and functional annotation of gene lists (2021 update). *Nucleic Acids Res*.
Stuart T, Butler A, Hoffman P, Hafemeister C, Papalexi E, Mauck WM, 3rd, Hao Y, Stoeckius M, Smibert P, Satija R. 2019. Comprehensive Integration of Single-Cell Data. *Cell* **177**: 1888-1902 e1821.
Thorvaldsdottir H, Robinson JT, Mesirov JP. 2013. Integrative Genomics Viewer (IGV): high-performance genomics data visualization and exploration. *Brief Bioinform* **14**: 178-192.
Virshup I, Rybakov S, Theis FJ, Angerer P, Wolf FA. 2021. anndata: Annotated data. *bioRxiv*: 2021.2012.2016.473007.

Supplemental Table S5. ATAC-seq primers with barcodes (Buenrostro et al., 2013).

Identifier	Sequence
Ad1 noMX	AATGATACGGCGACCACCGAGATCTACACTCGTCGGCAGCGTCAGATGTG
Ad2.1 TAAGGCGA	CAAGCAGAAGACGGCATACGAGATTCGCCTTAGTCTCGTGGGCTCGGAGATGT
Ad2.2 CGTACTAG	CAAGCAGAAGACGGCATACGAGATCTAGTACGGTCTCGTGGGCTCGGAGATGT
Ad2.3 AGGCAGAA	CAAGCAGAAGACGGCATACGAGATTTCTGCCTGTCTCGTGGGCTCGGAGATGT
Ad2.4 TCCTGAGC	CAAGCAGAAGACGGCATACGAGATGCTCAGGAGTCTCGTGGGCTCGGAGATGT
Ad2.5 GGACTCCT	CAAGCAGAAGACGGCATACGAGATAGGAGTCCGTCTCGTGGGCTCGGAGATGT
Ad2.6 TAGGCATG	CAAGCAGAAGACGGCATACGAGATCATGCCTAGTCTCGTGGGCTCGGAGATGT
Ad2.7 CTCTCTAC	CAAGCAGAAGACGGCATACGAGATGTAGAGAGGTCTCGTGGGCTCGGAGATGT
Ad2.8 CAGAGAGG	CAAGCAGAAGACGGCATACGAGATCCTCTCTGGTCTCGTGGGCTCGGAGATGT
Ad2.9 GCTACGCT	CAAGCAGAAGACGGCATACGAGATAGCGTAGCGTCTCGTGGGCTCGGAGATGT
Ad2.10 CGAGGCTG	CAAGCAGAAGACGGCATACGAGATCAGCCTCGGTCTCGTGGGCTCGGAGATGT
Ad2.11 AAGAGGCA	CAAGCAGAAGACGGCATACGAGATTGCCTCTTGTCTCGTGGGCTCGGAGATGT
Ad2.12 GTAGAGGA	CAAGCAGAAGACGGCATACGAGATTCCTCTACGTCTCGTGGGCTCGGAGATGT
Ad2.13 GTCGTGAT	CAAGCAGAAGACGGCATACGAGATATCACGACGTCTCGTGGGCTCGGAGATGT
Ad2.14 ACCACTGT	CAAGCAGAAGACGGCATACGAGATACAGTGGTGTCTCGTGGGCTCGGAGATGT
Ad2.15 TGGATCTG	CAAGCAGAAGACGGCATACGAGATCAGATCCAGTCTCGTGGGCTCGGAGATGT
Ad2.16 CCGTTTGT	CAAGCAGAAGACGGCATACGAGATACAAACGGGTCTCGTGGGCTCGGAGATGT
Ad2.17 TGCTGGGT	CAAGCAGAAGACGGCATACGAGATACCCAGCAGTCTCGTGGGCTCGGAGATGT
Ad2.18 GAGGGGTT	CAAGCAGAAGACGGCATACGAGATAACCCCTCGTCTCGTGGGCTCGGAGATGT

Supplemental Table S6. List of genes used to group DIV24 cells into three populations: cycling progenitors, transitional progenitors, and neurons.

Cycling Progenitors	Transitional Progenitors	Neurons
PAX6	*ASCL1*	*TUBB3*
FABP7	*SOX4*	*DCX*
VIM	*DLX1*	*ELAVL3*
NES	*DLL1*	*ELAVL4*
SOX2	*RGS16*	*MAP2*
MCM2	*GADD45G*	*BCL11B*
MKI67	*CDKN1C*	*RBFOX3*
TOP2A	*HES6*	*NEFM*
UBE2C	*SMOC1*	*STMN1*
SLC1A3	*DLL3*	*STMN2*
HES5	*IGFBP5*	
HES1		

Supplemental Table S7. Samples subsetted from Braun *et. al.* 2022 (https://github.com/linnarsson-lab/developing-human-brain) for label transfer to DIV24 WT DAPT (see Suppl. Fig. S1F)

Donor	Sample_ID	Age	Weeks	Dissected_region
XDD:348	10X177_4	5	5	Forebrain
XDD:348	10X177_5	5	5	Forebrain
XDD:348	10X177_6	5	5	Forebrain
XDD:348	10X178_1	5	5	Forebrain
XDD:400	10X298_1	5.5	5	Forebrain
XDD:400	10X298_2	5.5	5	Forebrain
XHU:305	10X111_1	7.5	7	Forebrain
XHU:305	10X111_2	7.5	7	Forebrain
XHU:305	10X112_1	7.5	7	Forebrain
XHU:305	10X112_2	7.5	7	Forebrain
XDD:313	10X132_1	8.5	8	Forebrain (one hemisphere)
XDD:313	10X132_2	8.5	8	Forebrain (one hemisphere)
XDD:313	10X132_3	8.5	8	Forebrain (one hemisphere)
XDD:313	10X132_4	8.5	8	Forebrain (one hemisphere)
XDD:313	10X132_5	8.5	8	Forebrain (one hemisphere)
XDD:313	10X132_6	8.5	8	Forebrain (one hemisphere)
XDD:313	10X132_7	8.5	8	Forebrain (one hemisphere)
XDD:313	10X132_8	8.5	8	Forebrain (one hemisphere)
XDD:313	10X143_1	8.5	8	Forebrain
XDD:313	10X143_2	8.5	8	Forebrain
XHU:307	10X114_3	9.2	9	Forebrain
XHU:307	10X114_4	9.2	9	Forebrain
XHU:307	10X115_4	9.2	9	Forebrain
XHU:307	10X115_5	9.2	9	Forebrain
XHU:307	10X115_6	9.2	9	Forebrain
XHU:292	10X92_1	9.5	9	Forebrain (cortex)
XHU:292	10X92_2	9.5	9	Forebrain (cortex)
XHU:297	10X101_5	10	10	Forebrain
XHU:297	10X101_6	10	10	Forebrain
XHU:297	10X101_7	10	10	Forebrain
XHU:297	10X101_8	10	10	Forebrain
XHU:297	10X102_5	10	10	Forebrain
XHU:297	10X102_6	10	10	Forebrain
XHU:297	10X102_7	10	10	Forebrain
XHU:297	10X102_8	10	10	Forebrain
